# Gut Microbiota is Associated with Aging‐Related Processes of a Small Mammal Species under High‐Density Crowding Stress

**DOI:** 10.1002/advs.202205346

**Published:** 2023-03-25

**Authors:** Xiaoming Xu, Guoliang Li, Da Zhang, Hanyi Zhu, Guang‐hui Liu, Zhibin Zhang

**Affiliations:** ^1^ State Key Laboratory of Integrated Management of Pest Insects and Rodents Institute of Zoology Chinese Academy of Sciences Beijing 100101 China; ^2^ University of Chinese Academy of Sciences Beijing 100049 China; ^3^ CAS Center for Excellence in Biotic Interactions University of Chinese Academy of Sciences Beijing 100049 China; ^4^ Institute for Stem cell and Regeneration CAS Beijing 100049 China; ^5^ State Key Laboratory of Membrane Biology Institute of Zoology Chinese Academy of Sciences Beijing 100101 China; ^6^ Beijing Institute for Stem Cell and Regenerative Medicine Beijing 100101 China

**Keywords:** anti‐aging, density‐dependent aging, gut microbiota, short‐chain fatty acids, social stress

## Abstract

Humans and animals frequently encounter high‐density crowding stress, which may accelerate their aging processes; however, the roles of gut microbiota in the regulation of aging‐related processes under high‐density crowding stress remain unclear. In the present study, it is found that high housing density remarkably increases the stress hormone (corticosterone), accelerates aging‐related processes as indicated by telomere length (in brain and liver cells) and DNA damage or inflammation (as revealed by tumor necrosis factor‐*α* and interleukin‐10 levels), and reduces the lifespan of Brandt's vole (*Lasiopodomys brandtii*). Fecal microbiota transplantation from donor voles of habitats with different housing densities induces similar changes in aging‐related processes in recipient voles. The elimination of high housing density or butyric acid administration delays the appearance of aging‐related markers in the brain and liver cells of voles housed at high‐density. This study suggests that gut microorganisms may play a significant role in regulating the density‐dependent aging‐related processes and subsequent population dynamics of animals, and can be used as potential targets for alleviating stress‐related aging in humans exposed to high‐density crowding stress.

## Graphical abstract

High‐density crowding stress can regulate the aging‐related processes through gut microorganisms. The elimination of high housing density or butyrate administration delays the appearance of aging‐related markers.

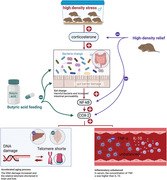

## 1 Introduction

Aging is a progressive and dynamic degenerative process, often accompanied with tissue stem cell depletion, tissue inflammation, cellular senescence, and metabolic dysfunction.^[^ ^1^ ^]^ Popular aging theories agree that the aging phenotype is caused by an imbalance between stressors and stress‐buffering mechanisms,^[^ ^2^ ^]^ and the loss of compensatory reserves leads to the accumulation of unrepaired DNA damage.^[^ ^3^ ^]^ Telomeres can be used as a typical marker of aging because their length reflects the replication potential of cells; furthermore, and telomeres are particularly vulnerable to oxidative damage.^[^ ^4^ ^]^ Persistent DNA damage can activate the tumor suppressor genes p53 and p21, and cause cell senescence.^[^ ^5^ ^]^ Inflammation is also considered one of the biological characteristics of aging.^[^ ^6^ ^]^ Nuclear factor‐kappa B (NF‐*κ*B) is the central component of cellular responses to damage, stress, and inflammation, and it is considered the most relevant transcription factor in mammalian aging.^[^ ^7^ ^]^ Chronic activation of the NF‐*κ*B‐cyclooxygenase‐2 (COX‐2)‐reactive oxygen species (ROS) axis results in accelerated aging in multiple tissues by the levels of affecting oxidative stress and inflammation.^[^ ^8^ ^]^ Therefore, the inhibition of NF‐*κ*B and COX‐2 activation can delay many aging‐related symptoms and rescue cell senescence.^[^ ^8^, ^9^ ^]^

Humans and animals are constantly exposed to various stresses that may accelerate their aging processes.^[^ ^10^ ^]^ Many external factors may influence aging processes, such as ionizing radiation,^[^ ^11^ ^]^ high temperature,^[^ ^12^ ^]^ and poor living environment.^[^ ^13^ ^]^ High‐density crowding is an important social stress factor in animals,^[^ ^14^ ^]^ which may accelerate their aging processes of humans and animals. In modern society, high‐density stress could lead to increased social stress as reflected by a subsequent increase in corticosterone (CORT) levels, leading to the appearance of premature aging features, for example, gray hair^[^ ^10a^ ^]^ or hair loss.^[^ ^15^ ^]^ People living in crowded urban areas usually have a higher probability of developing mental illnesses than those living in countryside areas.^[^ ^16^ ^]^ Repeated exposure to environmental stressors has been shown to shorten the lifespan of *Caenorhabditis elegans*.^[^ ^10b^ ^]^ An increase in population density can reduce the lifespan of *Drosophila melanogaster* (*D. melanogaster*).^[^ ^17^ ^]^ In nature, the variation in the population density of animals is largely driven by external (climate, food, and predators) and intrinsic (crowding and competing) factors. Density dependence is an important mechanism in regulating the population dynamics of animals. High density can increase social stress^[^ ^14^, ^18^ ^]^ and induce negative effects on immune function,^[^ ^18a^ ^]^ which may eventually contribute to the low survival rate of animals and a decline in their population.^[^ ^19^ ^]^ High‐density crowding stress could decrease the fitness of animals through various regulatory mechanisms such as the regulation of physiological,^[^ ^20^ ^]^ behavioral,^[^ ^21^ ^]^ genetic,^[^ ^22^ ^]^ senescence,^[^ ^19^ ^]^ neurology,^[^ ^14^, ^18^ ^]^ and gut microbes.^[^ ^14^, ^23^ ^]^ All these mechanisms are closely related to high‐density crowding stress. However, the density‐dependent aging processes of animals have not yet been fully evaluated in mammals.

Several studies have shown that gut microbiota is closely related to aging, and it can simultaneously affect a variety of aging processes, including inflammation^[^ ^24^ ^]^ and oxidative stress,^[^ ^25^ ^]^ which may affect ecological and evolutionary traits of hosts.^[^ ^26^ ^]^ Transplantation of gut microbiota from healthy young mice to elderly male mice attenuates behavioral deficits associated with aging.^[^ ^27^ ^]^ Several microorganisms (e.g., *Akkermansia muciniphila*, *Alistipes*, and *Prevotella*) have a close relationship with aging.^[^ ^28^ ^]^ Physiological and psychological stress can disrupt the composition of gut microbiota in humans and rodents^[^ ^29^ ^]^ and damage the intestinal permeability of mice.^[^ ^30^ ^]^ As a metabolic product of gut microbiota, butyric acid can regulate age‐related gut microbiota disorders and reverse aging‐related phenotypes.^[^ ^31^ ^]^ Although a few studies have indicated that high‐density crowding could increase social stress^[^ ^14^, ^18^ ^]^ and alter the composition of gut microbiota,^[^ ^14^, ^23^ ^]^ it remains unclear whether gut microorganisms themselves are involved in regulating the aging‐related processes of animals under high‐density conditions.

Brandt's vole (*Lasiopodomys brandtii*) is a typical social herbivore found in the steppe grasslands of Inner Mongolia, China. Adult voles weigh ≈30–40 g in autumn and 50 g in spring. They live in groups, and each family comprises two to seven breeders. They collectively defend their burrow systems. The overwintering voles breed two to three times a year from late April to early August, with a litter size of ≈2–15. The voles have a promiscuous mating system. The lifespan of a typical voles is ≈1‐year in field conditions, but it can extend up to 3‐years under laboratory conditions. The population of voles fluctuates periodically with a high density of ≈600–1000 voles per hectare in the field; this fluctuation is likely driven by both intrinsic factors (e.g., density‐dependence) and external factors (e.g., climate change).^[^ ^32^ ^]^ Our previous studies demonstrated that high‐population density increased aggressive behavior characterized by a decrease in oxytocin level but an increase in vasopressin (AVP) level in the brain, an increase in the serum CORT levels, and a significantly altered composition of gut microorganisms.^[^ ^14^, ^18^, ^23^ ^]^ The roles of gut microbiota play in aging‐related processes of Brandt's voles under high‐density crowding stress have never been investigated. Considering the known association of density with social stress and gut microbiota, in the present study, we focused on testing the following hypotheses: (1) high‐density crowding can increase social stress and accelerate aging‐related processes of voles, (2) implantation of gut microbiota of high‐density donor voles can cause a similar aging‐related process in recipient voles, and (3) interventions to mitigate high‐density crowding or butyric acid administration (to improve intestinal health) can alleviate the aging‐related processes of high‐density housed voles. The first hypothesis was tested by Experiment 1 using both male and female voles, while the second and the third hypothesis were tested using only male voles.

## 2 Results

### 2.1 Effects of Housing Density on the Lifespan of Voles

**Figure 1:**
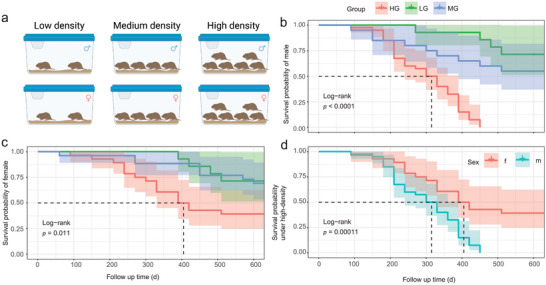
Effects of housing densities on the lifespan of Brandt's voles under laboratory conditions. a) Schematic diagram of the experimental design. b) Survival probability of male voles at different housed densities. c) Survival probability of female voles at different housed densities. d) Difference in survival probability between male and female voles housed under high‐density. Kaplan–Meier survival curves subjected to the log‐rank test. Benjamin–Hochberg was used to correct *p* value. LG, low‐density group, *n* = 14; MG, medium‐density group, *n* = 28; HG, high‐density group, *n* = 42. Voles that died due to fighting were excluded from data analysis.

In Experiment 1, housing densities showed significant effects on the lifespan of both male and female voles (as reflected by survival probability) for 630 days (male: *p* < 0.0001; female: *p* = 0.011). Compared to the male voles in the low‐density group (LG) and the medium‐density group (MG), those in the high‐density group (HG) showed significantly lower survival probability (LG: *p* < 0.0001; MG: *p* < 0.0001, **Figure** **1**b). Similarly, the survival probability of female voles in the HG was significantly lower than that of female voles in the LG and MG (LG: *p* = 0.041; MG: *p* = 0.04, Figure 1c). Female voles showed significantly higher survival probability at high‐density than male voles (*p* = 0.00011, Figure 1d).

### 2.2 Effects of Housing Density on Stress and Physiological Aging‐Related Indicators

**Figure 2:**
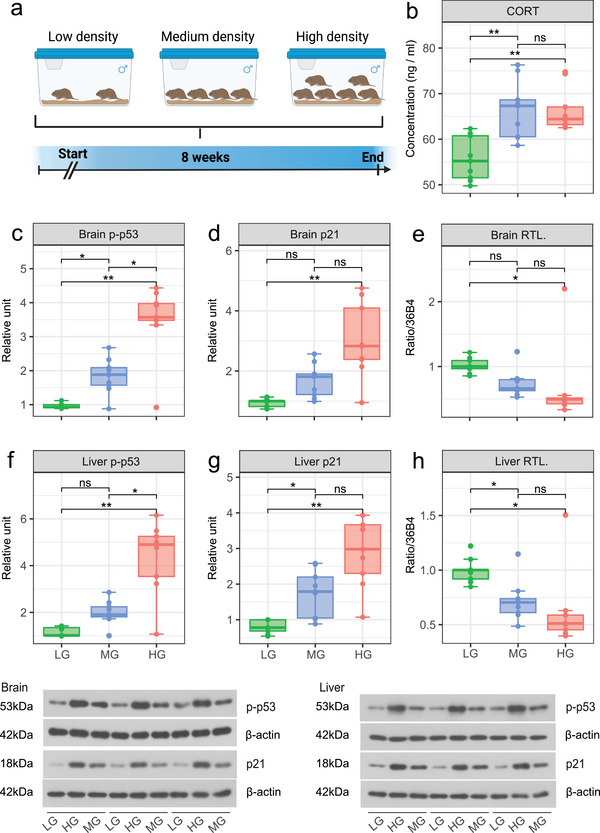
Effects of housing densities on stress level and aging‐related parameters of male Brandt's voles. a) Schematic diagram of the experimental design. b) Serum CORT level in voles at the end of week 8. c–e) Relative expression levels of phospho‐p53 and p21 and relative telomere length in brain cells. f–h) Relative expression levels of phospho‐p53 and p21 and relative telomere length in liver cells. For box plot, centerline: median; box limits: upper and lower quartiles; whiskers: two lines that go from the minimum to the lower quartile and then from the upper quartile to the maximum. A linear mixed model was used to determine the differences between groups, with groups as fixed factors and different cages as random factors. The “emmeans” function was used to perform the multiple comparisons test. * *p* < 0.05, ** *p* < 0.01, ns, not statistically significant. LG, low‐density group, *n* = 9; MG, medium‐density group, *n* = 9; HG, high‐density group, *n* = 9. RTL is the abbreviation for relative telomere length.

The results of Experiment 2 showed that stress level and physiological aging‐related indicators of male voles were significantly affected by different housing densities. As shown in **Figure** **2**b, the serum CORT level of voles in the MG (*F*_2, 35_ = 14, *p* = 0.004) and the HG (*p* = 0.002) was significantly higher than those housed in LG. In brain tissues, the expression level of p‐p53 (Figure 2c) in voles in the HG was significantly higher than those in the LG (*F*_2, 24_ = 33.14, *p* = 0.0017) and MG (*p* = 0.0269); furthermore, the expression level of p‐p53 in the MG was also significantly higher than those in the LG (*p* = 0.0351). In brain tissues, the expression level of p21 alone (Figure 2d) in voles in the HG was significantly higher than those in the LG (*F*_2, 24_ = 15.66, *p* = 0.0067). In liver tissues, the expression levels of both p‐p53 and p21 were significantly higher in the HG than in the LG (p‐p53: *F*_2, 24_ = 28.21, *p* = 0.0025, Figure 2f; p21: *F*_2, 24_ = 21.53, *p* = 0.0037, Figure 2g). The relative telomere length of the brain and liver cells of the HG was significantly smaller than that of in the LG (brain: *F*_2, 24_ = 4.24, *p* = 0.045, Figure 2e; liver: *F*_2, 24_ = 6.39, *p* = 0.031, Figure 2h). The expression level of claudin‐1 in the small intestines of the HG was significantly lower than that of the LG (*F*_2, 24_ = 4.73, *p* = 0.005, Figure S1k, Supporting Information). The serum of tumor necrosis factor‐*α* (TNF‐*α*) level and the liver super oxide dismutase (SOD) level in the LG were significantly lower than those in the MG (TNF‐*α*: *F*_2, 35_ = 14.69, *p* = 0.002; SOD: *F*_2, 8.8_ = 9.93, *p* = 0.008) and HG (TNF‐*α*: *p* = 0.002, Figure S1i, Supporting Information; SOD: *p* = 0.008, Figure S1d, Supporting Information). The interleukin‐10 (IL‐10) level in the HG was significantly higher than that in both the LG (*F*_2, 6.99_ = 16.36, *p* = 0.002) and MG (*p* = 0.019, Figure S1j, Supporting Information). No significant differences in the levels of 8‐hydroxy‐2 deoxyguanosine (8‐OHdG) (brain: *p* = 0.414; liver: *p* = 0.134), 4‐hydroxynonenal (4‐HNE) (brain: *p* = 0.2658; liver: *p* = 0.2967), and catalase (CAT) (brain: *p* = 0.8655; liver: *p* = 0.209) in the brain and liver were observed among the three density groups (Figure S1a,c,e–h, Supporting Information).

We determined the cognitive function of voles by using a Y‐maze, which requires voles to use learning and memory function to move toward a location which originally had food. Statistical analysis showed no significant differences in visit frequency to the food arm (*p* = 0.2482), the cumulative time in the food arm (*p* = 0.3282), the percentage of visit frequency in the food arm (*p* = 0.3282), and the percentage of the cumulative time in the food arm (*p* = 0.3372) between the three density groups of donor voles (Figure S2a–d, Supporting Information, all *p* > 0.05).

### 2.3 Effects of Housing Density on Gut Microbiome Diversity and Functional Genes

**Figure 3:**
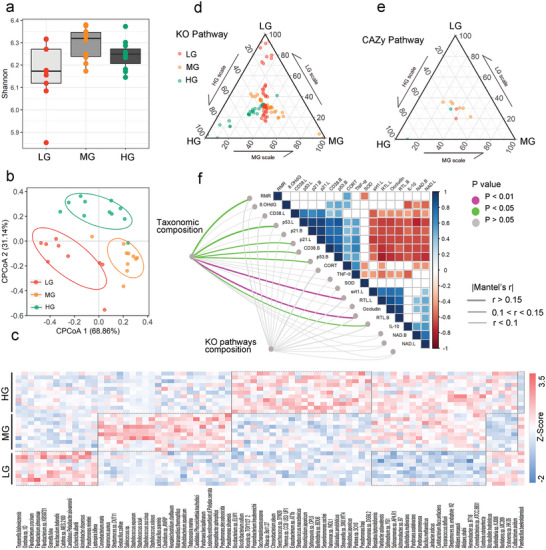
Effects of housing densities on the gut microbiome of male Brandt's voles . a) Differences in Shannon index represent differences in alpha diversity between the groups; Wilcoxon's method was used to analyze the differences. For box plot, centerline: median; box limits: upper and lower quartiles; whiskers: two lines that go from the minimum to the lower quartile and then from the upper quartile to the maximum. b) Constrained Principal Coordinate Analysis (CPCoA) plot of species‐level Bray–Curtis distances between samples from the LG, MG, and HG. c) The heatmap shows significantly different microbial species (*p* < 0.01) between the three different housing density groups. Species in the red part of the dotted boxes were enriched species and those in the blue part of the dotted boxes were depleted species. d,e) Ternary plots show differences in the functions of gut microbiome (d: KO function pathway; e: CAZy function pathway) for three housing densities. The significantly enriched pathways alone are shown. Each point corresponds to one specific function pathway. f) Pairwise relationship between aging‐related measurements with a color gradient denoting Pearson's correlation coefficient. Taxonomic and functional community structures were related to each physiological measurement by Mantel tests. Edge width denotes Mantel's *r* value for the corresponding distance correlations, and edge color indicates the corresponding statistical significance. B is the abbreviation of brain. L is the abbreviation for liver. RTL is the abbreviation for relative telomere length. LG, low‐density group, *n* = 9; MG, medium‐density group, *n* = 10; HG, high‐density group, *n* = 11.

In Experiment 2, we performed metagenomic shotgun sequencing for 33 fecal samples collected from the three density groups of male voles (11, 12, and 10 samples from the HG, MG, and, LG, respectively). A total of 6111 bacterial species were identified. Shannon index showed no significant differences between the three density groups (Wilcoxon rank‐sum test, *p*  =  0.36; **Figure** **3**a). The composition of gut microbiota was significantly affected by housing density (*F*_2, 29_ = 2.3, *p* = 0.02; Figure 3b). Based on the Bray–Curtis distance matrix, a distinct separation in the ordination space was observed for samples from the different housing density groups (Figure 3b). There were 13, 21, and 22 enriched species in the LG, MG, and HG, respectively. In contrast, the number of depleted species in the LG, MG, and HG was 18, 5, and 1, respectively (Figure 3c). The functional pathways of gut microbiota were also affected by housing density. We identified 114 important Kyoto Encyclopedia of Genes and Genomes (KEGG) Orthology (KO) pathways (61, 28, and 25 KO pathways enriched in the LG, MG, and HG, respectively) and 10 important Carbohydrate‐Active Enzyme (CAZy) function pathways (2, 6, and 2 pathways enriched in LG, MG, and HG, respectively) (Figure 3d,e; for details, see Tables S1 and S2, Supporting Information). The differential KO pathway (from Table S2, Supporting Information) was transformed into GO enrichment through the DAVID website. Gene Ontology (GO) enrichment showed that the biological processes of the HG and MG were enriched in the glycolytic process, while the biological process of the LG was enriched in cell redox homeostasis (Figure S3, Supporting Information).

To identify the overall relationship between gut microbiota and physiological aging indicators, the dissimilarities in gut microbial communities between individuals from each housing group were correlated with those in aging‐related measurements between individuals from each housing group. The Mantel test revealed that some indicators such as resting metabolic rate, p‐p53 and p21 protein expression levels in the liver, cluster of differentiation 38 and p‐p53 protein expression levels in the brain, sirtuin 1 protein expression level in the liver, occludin protein expression level, and relative telomere length of brain cells, were significantly associated with the composition of gut microbiota (all *p* < 0.05, Figure 3f). These physiological aging‐related indicators showed no significant relationship with the KO functional pathways (Figure 3f).

Linear discriminant analysis effect size (LEfSe) was used to screen bacterial species with significant differences in their relative abundance among the three groups (Figure S4, Supporting Information). *Akkermansia muciniphila* was significantly enriched in the LG. *Blautia producta* was significantly enriched in the MG. The genus *Alistipes* (e.g., *Alistipes shahii* [*A. shahii*], *A. dispar*, and *A. communis*) was significantly enriched in the HG.

We measured the concentrations of the following six short‐chain fatty acids in colonic feces: acetic acid, propionic acid, butyric acid, isobutyric acid, isovaleric acid, and valeric acid. The concentrations of propionic acid (*p* = 0.0144), butyric acid (*p* = 0.0081), isovaleric acid (*p* = 0.0269), and valeric acid (*p* = 0.0089) in the LG are higher than in the HG (Figure S5b,c,e,f, Supporting Information). The concentration of isobutyric acid showed no significant difference among the three groups (*p* = 0.3731, Figure S5d, Supporting Information).

### 2.4 Effects of Fecal Microbiota Transplantation (FMT) on Stress and Physiological Aging‐Related Indicators

**Figure 4:**
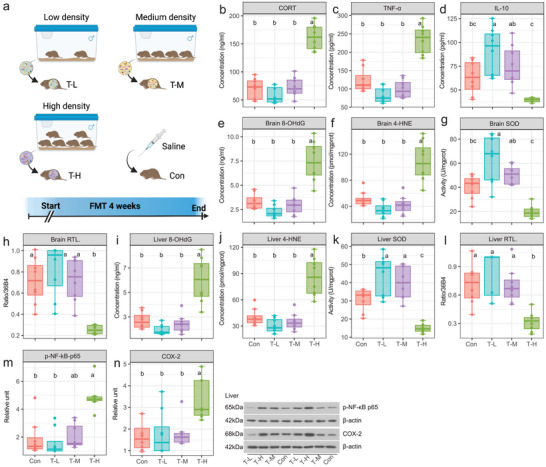
Effects of FMT of donor voles from the three density groups on the stress, oxidative damage, and relative telomere length of recipient voles. a) Schematic diagram of the experimental design. b–d) Serum CORT, TNF‐*α*, and IL‐10 levels of voles in different groups at the end of week 4. e–h) 8‐OHdG, SOD activity, and 4‐HNE levels and relative telomere length in brain. i–l) 8‐OHdG, SOD activity, and 4‐HNE levels and relative telomere length in liver. m,n) p‐NF‐*κ*B p65 and COX‐2 expression levels in liver. For box plot, centerline: median; box limits: upper and lower quartiles; whiskers: two lines that go from the minimum to the lower quartile and then from the upper quartile to the maximum. T‐L, Recipient voles with FMT from low‐density group (LG), *n* = 8; T‐M, Recipient voles with FMT from medium‐density group (MG), *n* = 8; T‐H, Recipient voles with FMT from high‐density group (HG), *n* = 8; Con, Voles with saline group, *n* = 8. One‐way analysis of variance was used to determine the difference between groups, and Tukey test was used for post hoc. Bars that do not share the same letter are significantly different from one another.

In Experiment 3, the serum CORT and TNF‐*α* level of the T‐H (i.e., recipient voles transplanted with fecal microorganisms from donor voles of the HG) were significantly higher than those of the T‐L (i.e., recipient voles transplanted with fecal microorganisms from donor voles of the LG) (**Figure** **4**b,c). The serum IL‐10 level of the T‐H was significantly lower than that of the T‐L (Figure 4d). The serum 8‐OHdG and 4‐HNE levels of the T‐H were significantly higher than those of the Con, T‐M (i.e., recipient voles transplanted with fecal microorganisms from donor voles of the MG) and T‐L (Figure 4e,f,i,j). In contrast, the tisssues levels of CAT (Figure S6a,b, Supporting Information) and SOD and the relative telomere length of brain and liver cells in the T‐H were significantly lower than those in the Con, T‐M, and T‐L (Figure 4g,k). p‐NF‐*κ*B p65 and COX‐2 expression levels in the liver cells of T‐H were significantly higher than those of the T‐L (Figure 4m,n). The expression level of claudin‐1  in the small intestine of the T‐H was significantly lower than that of the T‐L (Figure S6c, Supporting Information). The expression levels of p‐p53 and p21 in the brain cells of T‐H were alone significantly higher than those of the T‐L (Figure S6d,e, Supporting Information). The expression levels of p‐p53 and p21 in the liver cells of the T‐H were alone significantly higher than those of the T‐L and Con (Figure S6f,g, Supporting Information). Table S3, Supporting Information, shows detailed *p*‐values of the different treatment groups.

The results of the Y‐maze test showed no significant differences in visit frequency to the food arm, the percentage of visit frequency in the food arm between the three density groups of recipient voles (Figure S7a,c, Supporting Information, all *p* > 0.05). However, the cumulative time in the food arm and percentage of cumulative time in the food arm of the T‐H was significantly higher than those the T‐L (Figure S7b,d, Supporting Information, all *p* < 0.05), indicating T‐H voles had a higher capacity of memory and cognition.

### 2.5 Effects of FMT on Gut Microbiome Diversity and Functional Genes

**Figure 5:**
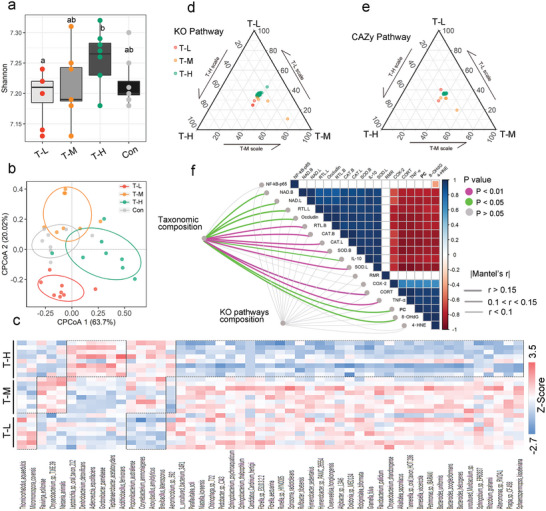
Effects of FMT of donor voles from the three density groups on the gut microbiome of recipient voles. a) Differences in Shannon index represent differences in alpha diversity between the groups; Wilcoxon's method was used to analyze the differences. For box plot, centerline: median; box limits: upper and lower quartiles; whiskers: two lines that go from the minimum to the lower quartile and then from the upper quartile to the maximum. b) CPCoA plot of species‐level Bray–Curtis distances between samples from the Con, T‐L, T‐M, and T‐H. c) The heatmap shows significantly different microbial species (*p* < 0.01) between the three different housing density groups. Species in the red part of the dotted boxes were enriched species and those in the blue part of the dotted boxes were depleted species. d,e) Ternary plots show differences in the functions of gut microbiome (d: KO function pathway; e: CAZy function pathway) for three housing densities. The significantly enriched pathways alone are shown. Each point corresponds to one specific function pathway. f) Pairwise relationship between aging‐related measurements with a color gradient denoting Pearson's correlation coefficient. Taxonomic and functional community structures were related to each physiological measurement by Mantel tests. Edge width denotes Mantel's *r* value for the corresponding distance correlations, and edge color indicates the corresponding statistical significance. B is the abbreviation of brain. L is the abbreviation for liver. RTL is the abbreviation for relative telomere length. PC is the abbreviation of protein carbonyl. T‐L, Recipient voles with FMT from low‐density group (LG), *n* = 8; T‐M, Recipient voles with FMT from medium‐density group (MG), *n* = 8; T‐H, Recipient voles with FMT from high‐density group (HG), *n* = 8; Con, Voles with saline group, *n* = 8.

The results of Experiment 3 showed that FMT significantly affected the alpha diversity of the gut microbiota of recipient voles (**Figure** **5**a). Shannon index in the T‐L was significantly lower than that in the T‐H (Wilcoxon rank‐sum test, *p* < 0.05; Figure 5a). A CPCoA of the taxonomic composition of fecal samples showed a clear separation between the FMT groups based on the Bray–Curtis distance (*F*_3, 31_ = 2.17, *p* = 0.01; Figure 5b). There were 2, 3, and 6 enriched species in the T‐L, T‐M, and T‐H, respectively. In contrast, the T‐L, T‐M, and T‐H were 4, 1, and 35 depleted species, respectively (Figure 5c). The functional pathways of gut microbiota were also affected by FMT. We identified 42 key KO pathways (3, 4, and 35 KO pathways enriched in the T‐L, T‐M, and T‐H, respectively) and 19 key CAZy function pathways (2, 4, and 13 CAZy function pathways enriched in the T‐L, T‐M and T‐H, respectively) (Figure 5d,e; for details, see Tables S4 and S5, Supporting Information). The differential KO pathway (from Table S5, Supporting Information) was transformed into GO enrichment through the DAVID website. GO enrichment showed that the biological processes of the T‐H were enriched in the sterol biosynthetic process and pyruvate metabolic process, while no such enrichment processes were observed in the T‐L and T‐M (Figure S8, Supporting Information). Stress hormone levels and some physiological aging indicators (CORT, RTL, CAT, NAD^+^, SOD, TNF‐*α*, IL‐10, 8‐OHdG, PC, and occludin) were significantly correlated with gut microbial composition (all *p* < 0.05) (Figure 5f).

LEfSe was used to screen bacterial species with significant differences in relative abundance among the Con, T‐L, T‐M, and T‐H. (Figure S9, Supporting Information). *Clostridioides difficile* (*C. difficile*), *Roseburia intestinalis* (*R. intestinalis*), and *Clostridium hylemonae* were significantly enriched in the T‐H. The genus *Muribaculum* (*Muribaculum* sp. H5, *M*. sp. TLL A4, and *M. intestinale*) was significantly enriched in the T‐L. *Prevotella dentalis*, *P. denticola*, and *Bacteroides dorei* were significantly enriched in the T‐M. In the Con, *Bacteroides* (*Bacteroides salanitronis*, *B. fragilis*, and *B. viscericola*) was significantly enriched.

### 2.6 Effects of Interventions on Stress and Physiological Aging‐Related Indicators

**Figure 6:**
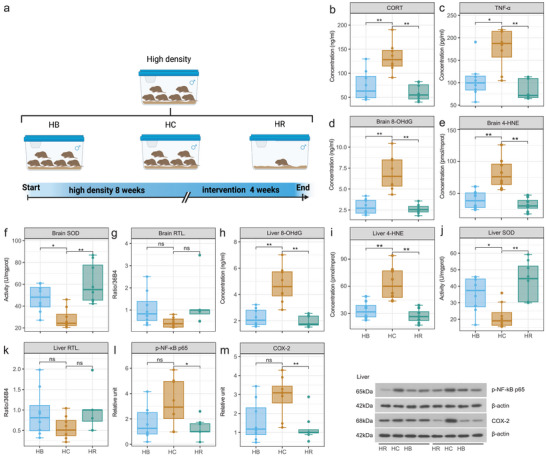
Effects of different intervention methods on alleviating oxidative damage and inflammation in high‐density voles. a) Schematic diagram of the experimental design. b,c) Serum CORT and TNF‐*α* levels of voles in different interventions group at the end of week 4. d–g) 8‐OHdG, 4‐HNE levels and SOD activity levels and relative telomere length in brain. h–k) 8‐OHdG, 4‐HNE levels, and SOD activity levels and relative telomere length in liver. l,m) p‐NF‐*κ*B p65 and COX‐2 expression levels in the liver. For box plot, centerline: median; box limits: upper and lower quartiles; whiskers: two lines that go from the minimum to the lower quartile and then from the upper quartile to the maximum. HB, high‐density butyric acid feeding group, *n* = 8; HR, high‐density relief group, *n* = 8; and HC, high‐density continuation group (i.e., no high‐density relief), *n* = 8. The statistical analysis in (a)–(m) was performed using two‐tailed unpaired *t*‐test. HC was included as control. * *p* < 0.05, ** *p* < 0.01, ns: not statistically significant.

In Experiment 4, we evaluated the effects of two intervention methods in alleviating social stress and physiological aging‐related indicators of high‐density voles. The serum CORT and TNF‐*α* levels were significantly lower in the HR (i.e., high‐density relief group) and HB (i.e., administration of butyric acid group) than in the HC (i.e., high‐density continuation group) (both *p* < 0.05; **Figure** **6**b,c). The levels of 8‐OHdG (both *p* < 0.05; Figure 6d,h) and 4‐HNE (both *p* < 0.05; Figure 6e,i) in the brain and liver and p‐NF‐*κ*B p65 and COX‐2 level in the liver were significantly lower in the HR than in the HC (both *p* < 0.05; Figure 6l,m). The SOD level in the brain and liver were significantly higher in the HR than in the HC (both *p* < 0.05; Figure 6f,j). Neither intervention had a significant effect on the relative telomere length of the liver and brain cells (*p* > 0.05; Figure 6g,k). Compared to the HC, the HB showed significantly increased SOD (both *p* < 0.05; Figure 6f,j) and CAT (both *p* < 0.05; Figure S10a,d, Supporting Information) levels in the brain and liver, and decreased levels of 4‐HNE (Figure 6e,i) in the brain and liver. Both interventions significantly increased the serum IL‐10 level (both *p* < 0.05; Figure S10h, Supporting Information). HR alone significantly decreased the expression levels of p‐p53 and p21 in the brain and liver cells (Figure S10b,c,e,f, Supporting Information; all *p* < 0.05). Administration of butyric acid significantly increased p21 expression in the brain (*p* < 0.05, Figure S10c, Supporting Information). Neither intervention had a significant effect on claudin‐1 expression in the small intestine (Figure S10g, Supporting Information, *p* > 0.05). Table S6, Supporting Information, shows detailed *p*‐values.

Regarding the cognitive function of voles, HR significantly increased the visit frequency to the food arm (*p* = 0.003); however, HB had no significant effect on the visit frequency to the food arm (Figure S11a, Supporting Information, *p* = 0.078). In contrast, HB significantly increased the percentage of cumulative time in the food arm (*t* = 2.781, *p* = 0.014); however, HR had no significant effect on the percentage of cumulative time in the food arm (Figure S11d, Supporting Information, *t* = 0.5635, *p* = 0.582). Neither of these two interventions, however, had significant effects on the cumulative time in the food arm (HR: *t* = 0.5303, *p* = 0.6042; HB: *t* = 1.590, *p* = 0.1341; Figure S11b, Supporting Information) and the percentage of visit frequency to the food arm (HR: *t* = 0.9728, *p* = 0.3472; HB: *t* = 1.747, *p* = 0.1025; Figure S11c, Supporting Information). These results suggested HR and HB would improve the ability of memory and cognition of high‐density voles to some extent.

## 3 Discussion

Our study provides clear evidence that high‐density crowding accelerates the aging process of Brandt's voles, which is manifested by shortening of relative telomere length and an increase in DNA damage. We also found that “high‐density microbiota” promotes the increase of aging‐related phenotype in voles. HR and administration of HB approaches alleviated the social stress and aging processes. Our results suggest that gut microbiota plays a significant role in mediating the aging‐related processes of voles under high‐density conditions, and can be used as a potential therapeutic target for treating stress‐related diseases in humans.

### 3.1 Effects of High‐Density Crowding on Aging‐Related Processes and Lifespan

Previous studies have shown that high‐density crowding would shortens the life‐span of *D. melanogaster* ^[^ ^17^ ^]^ and Zebra finches;^[^ ^33^ ^]^ however, but such observations are lacking in mammals. In the present study, we found that high housing density significantly shortened the lifespan of Brandt's voles. Under natural conditions, voles have a lifespan of ≈1 year, but they may survive to a maximum of ≈3 years in laboratory conditions (personal observation). Because we minimized the effects of direct fighting on the mortality of voles, the observed changes in lifespan in this study should mostly represent the natural aging processes of the voles. Therefore, accelerated aging in high density could be an important mechanism for regulating the population dynamics of animals,^[^ ^19^ ^]^ probably because of an increase in social stress due to crowding.^[^ ^18b^ ^]^

We found the survival time of female voles in HG is significantly longer than that of male voles. There have been many reports on discrepancies between the lifespans of female and male animals.^[^ ^34^ ^]^ For example, Lemaître et al. found that female mammals, including humans, live significantly longer than male mammals.^[^ ^35^ ^]^ The influence of chronic stress on inflammatory, oxidative, and cortisol responses may vary by gender, producing differences in telomere attrition between men and women.^[^ ^36^ ^]^ Powell‐Wiley et al. found that telomere declines were more pronounced in men than in women in higher deprivation settings.^[^ ^37^ ^]^ High‐density can increase aggression behavior and stress hormone (CORT) of Brandt's voles,^[^ ^14^, ^38^ ^]^ but evidence on female vole is rare. In field condition, there are very fewer males but more females in a burrow system or a family,^[^ ^38^, ^39^ ^]^ likely due more furious fighting in male voles. Zhang et al. found male Brandt's voles had a higher duration of sustained aggression than female Brandt's voles under high housing density.^[^ ^39b^ ^]^ These observations may explain the lower survival rate of male voles than female voles. Different housing density can significantly affect the diversity of gut microbes of both male and female Brandt's voles,^[^ ^23^ ^]^ which may be associated with the gender difference in life span. Unfortunately, we have no data on the effect of high‐density housing on aging‐related processes and gut microbes for female voles, which need further investigation in future.

High‐density crowding can increase social stress (i.e., CORT level) and then likely accelerate aging in both humans^[^ ^10^, ^15^ ^]^ and animals.^[^ ^18b^ ^]^ Long‐term CORT administration caused premature aging in rats.^[^ ^40^ ^]^ Our results showed that the serum CORT level of the HG was significantly higher, which is consistent with previous observations^[^ ^14a^ ^]^ and supports our first hypothesis. Therefore, an increase in the social stress‐related hormone in high‐density voles may be a driving factor in accelerating aging‐related processes.

Previous studies have shown that stress or harsh living conditions can shorten telomere length.^[^ ^41^ ^]^ The transcription factor p53 is activated by telomere attrition and oxidative DNA damage.^[^ ^42^ ^]^ In p53‐dependent premature aging, cell cycle arrest is regulated by p21.^[^ ^43^ ^]^ In the present study, we found that the HG had the shortest relative telomere length among the three density groups, and high density was associated with p‐p53 and p21 overexpression in the brain and liver of voles.Therefore, we believe that high‐density crowding stress may promote the aging‐related process through the regulation of p‐p53 and p21.

8‐OHdG is one of the most common oxidative DNA damage markers, and its cumulative amount in many tissues increases with age.^[^ ^44^ ^]^ As a lipid peroxidation product, 4‐HNE is considered a marker of aging‐related oxidative stress.^[^ ^45^ ^]^ In the present study, high density had no significant effect on 8‐OHdG and 4‐HNE level in donor voles; however, differences in 8‐OHdG and 4‐HNE levels were observed in FMT recipient voles. High‐density crowding caused a significantly increased and decreased serum TNF‐*α* and IL‐10 levels, respectively. As reported previously, chronic inflammation is related to aging.^[^ ^6^ ^]^ This may be because of the increased permeability of the intestinal barrier (downregulated expression of claudin‐1) by the disrupted gut microbial composition under high‐density stress.^[^ ^30^ ^]^ This leads to the leakage of microorganisms and their metabolites through the intestinal barrier, subsequently resulting unnecessary inflammation^[^ ^46^ ^]^ and DNA damage.^[^ ^47^ ^]^ Our results showed that high density increased intestinal permeability and caused an imbalance between pro‐inflammatory and anti‐inflammatory factors, which might accelerate the aging‐related processes of voles.

### 3.2 Association of Gut Microbiota with Stress and Aging‐Related Indicators under High‐Density Conditions

*A. muciniphila* was enriched in low‐density voles. Previous studies have indicated that the abundance of *A. muciniphila* in aged mice was low, and their involvement in the butyric acid synthesis pathway was reduced.^[^ ^28a^ ^]^ Oral administration of *A. muciniphila* can improve the intestinal phenotype of aging mice and prolong their healthy lifespan.^[^ ^28b^ ^]^ An increase in the abundance of *Prevotella* is a sign of aging in cynomolgus monkeys.^[^ ^28c^ ^]^ Reducing the relative abundance of *Alistipes* and *Prevotella* could be an anti‐aging approach.^[^ ^28d^ ^]^ In our present study, *Alistipes* (e.g., *A. shahii*, *A. dispar*, and *A. communis*) and *Prevotella* were found to be enriched in the HG. The relative abundances of *Propionibacterium freudenreichii* and *Streptococcus macedonicus*, which are probiotics for anti‐aging, were relatively lower in HG voles.^[^ ^48^ ^]^ Gut microbiota may compensate for or support the aging process of the body through positive stimulation.^[^ ^49^ ^]^ Both LG and MG had more abundant bacteria that produce short‐chain fatty acids, thereby reducing inflammation and maintaining the stability of gut microbiota. For example, *Blautia* and *Eubacterium* can produce acetic acid^[^ ^50^ ^]^ and butyric acid.^[^ ^51^ ^]^ Notably, in the assay of fecal short‐chain fatty acids, we also found significantly higher concentrations of acetic acid, propionic acid, and butyric acid in the feces of the LG than in the feces of the HG. Butyric acid is beneficial for anti‐aging.^[^ ^49^ ^]^ Aging is often associated with a reduction in the abundance of *Akkermansia* and butyric acid‐producing bacteria, and damage to the gut integrity.^[^ ^31a^ ^]^ Our results suggest that this specific anti‐aging or anti‐inflammatory or probiotic bacterial species might play an important role in regulating density‐dependent aging processes of voles.

### 3.3 Gut Microbiota May Mediate Aging‐Related Processes under High Density

Recent studies indicated that FMT from young mice to old mice can reverse the aging characteristics of the intestine, eyes, and brain. Conversely, FMT from old mice to young mice will cause inflammation of the brain and eyes.^[^ ^52^ ^]^ It remains unclear whether “high‐density microbiota” could accelerate the aging‐related processes of animals through FMT. In the present study, we found that “high‐density microbiota” induced similar changes in the levels of CORT, and claudin‐1 in the small intestine, p‐p53 and p21 expression levels, relative telomere length in brain and liver cells, antioxidative level (SOD), and inflammatory imbalance (serum TNF‐*α* and IL‐10 levels) in recipient voles. Our results demonstrated that gut microorganisms could play a significant role in regulating the aging‐related processes of animals.

A slight difference in aging‐related indicators in response to density treatment was observed between donor voles (Experiment 2) and recipient voles (Experiment 3). Although 8‐OHdG and 4‐HNE levels showed a non‐significant difference in the different density groups of donor voles, these levels were significantly higher in the T‐H (Table S3, Supporting Information). This difference suggested an imbalance between oxidation and antioxidation in T‐H voles (an increase in 8‐OHdG and 4‐HNE levels and a decrease in SOD and CAT levels). The disrupted composition of gut microorganisms could be a key factor in causing the imbalance between oxidation and antioxidation. Alterations in the gut microbial composition may induce inflammation and oxidative damage due to an increase in NF‐*κ*B expression, or activation of NF‐*κ*B, which upregulates COX‐2.^[^ ^53^ ^]^ Chronic activation of the NF‐*κ*B‐COX‐2‐ROS axis leads to accelerated aging.^[^ ^8^ ^]^ Our results showed that transplantation of gut microbiota from HG donor voles significantly upregulated NF‐*κ*B and COX‐2 expression levels in recipient voles, thus supporting our second hypothesis. Gut microbiota may promote the aging‐related process by enhancing NF‐*κ*B and COX‐2 pathway‐mediated oxidative DNA damage under high density. This also implies that maintaining gut microbiota homeostasis and inhibiting DNA damage could be an effective intervention strategy.

Previous studies have reported that *Adlercreutzia* has both harmful and beneficial effects.^[^ ^54^ ^]^ In our present study, *Adlercreutzia* abundance was higher in both the HG and T‐H, thus suggesting *Adlercreutzia* could increase aging in high density. *C. difficile* was significantly enriched in the T‐H, which may also enhance aging because inhibition of *C. difficile* is beneficial to healthy aging.^[^ ^55^ ^]^ A notable finding was that a large number of butyric acid‐producing bacteria were found in the T‐H, such as *R. intestinalis, Butyrivibrio*, *and Eubacterium rectale* (*E. rectale*). Gut microbiota may adapt to reverse physiological changes of a host.^[^ ^56^ ^]^ The enrichment of butyric acid‐producing bacteria in T‐H could be used to repair DNA damage or inflammation in high‐density conditions; a similar intervention relief effect was observed in Experiment 4 (see below). Furthermore, the abundance of *Leptotrichia_*sp*._*oral_taxon_212 and *Gordonibacter_pamelaeae* increased in T‐H voles, whereas the abundance of *Bacteroides_uniformis* decreased. *Leptotrichia_*sp.*_*oral_taxon_212 plays a role in causing several infections, and it is an opportunistic pathogen that can cause several negative effects on hosts with low immunity.^[^ ^57^ ^]^ *Gordonibacter_pamelaeae* can also cause several infections, including bacteremia^[^ ^58^ ^]^ and is related to Crohn's disease. *Bacteroides_uniformis* can enhance the intestinal barrier and reduce inflammation.^[^ ^59^ ^]^ The increase in the abundance of these pathogens and the decrease in the proportion of anti‐aging bacteria are likely to be one of the factors responsible for increasing the oxidative damage and including a proinflammatory response in high‐density voles.

Donor and recipient voles had different treatment environments, which would result in difference in gut microbiota (Figures 3a and 5a) and GO enrichment result (Figures S1 and S8, Supporting Information) in response to housing density. Donor voles experienced stress of high‐density housing directly by social contact and psychological perception through hypothalamic‐pituitary‐adrenal (HPA) axis. In contrast, the recipient voles experienced stress of high‐density housing directly by receiving gut microbiota and fecal metabolites of donor voles. In addition, the choice of pre‐treatment with antibiotics or not was also a key reason for the above response difference between donor and recipient voles. Our previous study has shown that pre‐treatment for recipient voles might also modify the GO pathways by altering the gut microbiota.^[^ ^60^ ^]^ We found that increased oxidative damage and inflammation in HG voles is associated with the enriched glycolysis, which is consistent with previous studies that increased oxidative stress and increased inflammation can promote glycolysis of the host.^[^ ^61^ ^]^ The biological process of sterol synthesis in T‐H voles after FMT is significantly different from HG voles. Sterol synthesis is well known to be closely related to health of animals and people; for example, high‐level cholesterol can cause various diseases (e.g., cardiovascular disease)^[^ ^62^ ^]^ and then may promote aging‐related processes.^[^ ^63^ ^]^ Many bacteria can synthesize sterols, and sterols produced by bacteria are different from those produced by vertebrates.^[^ ^64^ ^]^ Pre‐antibiotics treatment may affect the community structure of gut microbiota by shaping the direction of priority effect and helping colonization of FMT gut microbiota from high‐density housing group, and then facilitate the bacterial sterols synthesis and result in significant higher Shannon diversity index in T‐H voles (Figures 3a and 5a and Figures S1 and S8, Supporting Information).

### 3.4 Effects of Stress Interventions on Aging‐Related Processes

Several studies have shown that aging induced by stress is reversible. For example, stress relieving could help in replacing gray hair with black hair, and individual emotional regulation and self‐control can alleviate aging‐related stress in younger populations.^[^ ^9^, ^65^ ^]^ Our present study also showed that the HR approach significantly reduced social stress and alleviated the aging‐related processes of the liver. HB and HR approach also significantly improved memory function in Brandt's voles. Previous studies reported that butyrate can help elderly mice combat some of the debilitating effects of aging and to delay aging.^[^ ^66^ ^]^ In present study, we provide new evidence that butyric acid supplementation can alleviate the aging symptoms caused by high‐density crowding, supporting our third hypothesis.

Although both HB and HR approach effectively relieved the aging‐related process of voles caused by high‐density stress, the mechanism may differ between them. Because organ aging is asynchronous,^[^ ^67^ ^]^ the response of different organs to aging intervention may vary. Our results showed that HR had a strongly downregulated the expression of p‐p53 and p21 as compared to HB. HB upregulated the SOD level, thus suggesting that the mechanism by which butyric acid relieve accelerated aging may involve its antioxidative property. Alleviating aging‐related processes through HR intervention may be mediated by the normalization of proinflammatory levels and the activity of the HPA axis. Notably, both HB and HR interventions downregulated COX‐2 expression, thus suggesting that they may affect DNA damage by modulating COX‐2 expression levels and ameliorating the imbalance caused by oxidative damage. Thus, it is possible to suppress the stress‐driven aging‐related process by restoring the stability of gut microbiota (i.e., increasing the proportion beneficial bacteria and decreasing the proportion of harmful bacteria) and inhibiting the upregulation of NF‐*κ*B and COX‐2 expression levels. It is necessary to choose the integrated intervention methods to effectively alleviate the adverse health consequences of stress.

## 4 Conclusion 

**Figure 7:**
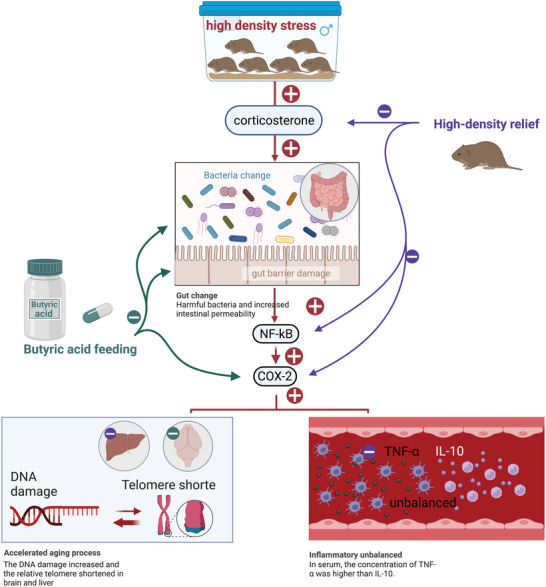
Schematic diagram summarizing the effects and potential mechanism of high‐density crowding stress and interventions (high‐density relief, and butyric acids administration) on aging‐related processes in Brandt's voles. Different colors are used to show the different influencing factors. The red arrow and plus sign represent the role of high‐density stress. The green arrow and minus sign represent the role of butyric acid administration. The purple arrow and minus sign represent the role of high‐density relief. The direction of the arrow indicates the target molecule or organ of action. A plus or minus sign implies to promotion or inhibition, respectively.

In summary, high‐density crowding can shorten the lifespan of individuals, probably through aging‐related physiological processes. High density increased the level of the stress hormone CORT, which disrupted the composition of gut microbiota by decreasing the abundance of anti‐aging or anti‐inflammatory bacterial species and increasing the proportion of pathogenic bacteria, thereby causing an increase in DNA oxidation and inflammation through upregulation of the NF‐*κ*B and COX‐2 pathways (**Figure** **7**). HR intervention alleviated the aging‐related processes by reducing stress levels and inhibiting the NF‐*κ*B and COX‐2 pathways, while HB intervention alleviated the aging‐related processes by improving gut health and downregulating the expression of NF‐*κ*B and COX‐2 in high‐density voles (Figure 7).

Our study also has several limitations. First, our study mostly focused on the physiological processes of aging; however, behavioral or morphological aging phenotypes were not fully evaluated. Second, although FMT from HG donor voles could produce similar aging‐related processes in recipient T‐H voles, the underlying mechanisms of the identified microorganisms or pathways remain unclear. Third, although HR and HB intervention could reverse the aging‐related processes of adult voles, it remains unclear whether they could reverse the aging process in terms of lifespan. Future studies should identify the key bacterial and their metabolites that play a role in regulating the density‐dependent aging processes in animals. Finally, the result on males may be different from females. The reason we did not choose females is that females have about 1 week estrus cycle which would alter their hormone levels and then gut microbiota. Thus, using female would bring uncertainty or larger variation to the effects of treatments. Males are more homogeneous than females in physiology during the study period. Besides, males would suffer more stress than females in high density because males are more aggressive than females. It is expected that the crowding effect of high density on females should be smaller than males because females are more tolerant to each other living in a burrow system.^[^ ^36^, ^39^ ^]^

Our study may have some implications for managing human health or animal populations. People in big city with high population density may suffer high social stress and high risk of developing mental illness,^[^ ^68^ ^]^ for example, psychosis and depression,^[^ ^69^ ^]^ and then have short telomere length.^[^ ^70^ ^]^ The gut microbiota could be used as a potential therapeutic target to alleviate diseases symptoms related to human stress. The inhibition of pathogenic bacteria (e.g., *Alistipes*, *C. difficile, Leptotrichia_*sp._oral_taxon_212, and *Gordonibacter_pamelaeae*) or the administration of probiotic bacteria (e.g., *Bacteroides_uniformis* and *A. muciniphila*) or short‐chain‐fatty acid‐producing bacteria (e.g., *R. intestinalis, Butyrivibrio*, and *E. rectale*)^[^ ^71^ ^]^ could help to resist and improve decline in health. Additionally, mitigation of stress through appropriate relaxation methods could be an effective approach to alleviate high‐density aging. The population of Brandt's voles erupts every 5–7 years, which often causes huge damage to the grassland.^[^ ^72^ ^]^ Harmful or pathogenic bacteria could be selected for reducing population outbreaks of voles in the grassland. Further work is needed to assess the feasibility of these gut microorganisms in managing human health or animal populations in the future.

## 5 Experimental Section

### Experimental Animals

Adult Brandt's voles of these experiments were collected from a laboratory colony at the Institute of Zoology, Chinese Academy of Sciences in Beijing, China. Adult age could be the turning point toward old age, thus, adult voles were selected for testing the crowding effect of high density on aging‐related processes of voles. They were raised at polyethylene plastic cages (30 × 15 × 20 cm) with corn cob as bedding material and provided with rabbit pellets (containing 18% protein, 3% fat, 12% fiber, and 47% carbohydrate, Beijing KeAo Bioscience Co.) and water ad libitum. All experimental voles were maintained in a room with temperatures of 23 ± 0.5 °C and a photoperiod of 14Light:10Dark (lights turned on at 06:00). All animals handling and raising were conducted in line with guidelines Animal Care and Use Committee of Institute of Zoology, Chinese Academy of Sciences.

### Experimental Design

*Experiment 1*: This experiment was conducted to determine the effects of housing density on the lifespan of voles. 9‐month‐old male or female voles were divided into the LG (two voles per cage, cage numbers = 7), the MG (four voles per cage, cage numbers = 7), and the HG (six voles per cage, cage numbers = 7) (Figure 1a). The authors’ previous studies indicated that these three housing density levels could produce an apparent gradient of social stress.^[^ ^14^, ^18^ ^]^ To eliminate the effects of reproduction, the experiments for males and females were conducted separately. Voles were selected from the same family to minimize mortality due to serious fighting during long‐term cohabitation (very few voles from different families could survive together). Voles that died due to fighting‐related injuries were excluded from the analysis.

*Experiment 2*: This experiment was performed to test the effects of housing density on aging‐related processes as reflected by aging markers of the brain and liver in Brandt's voles. 4‐month‐old male voles (*n* = 34, average weight = 39.55 g) were randomly assigned to three different housing density groups (Figure 2a): low‐density group (LG, two voles per cage, *n* = 10), medium‐density group (MG, four voles per cage, *n* = 12), and high‐density group (HG, six voles per cage, *n* = 12). Voles in each cage originated from different families and were born on the same day. Voles from different families were selected to simulate high social stress in a short experimental period of a few weeks.^[^ ^14^, ^18^, ^23^ ^]^ The experimental voles were raised in a laboratory environment for 8 consecutive weeks. At the end of the experiment, voles were sacrificed, and their feces, serum, tissues, and organs were collected immediately for further analysis.

*Experiment 3*: To investigate the potential roles of gut microbiota in mediating housing density‐induced effects on aging‐related processes, an FMT experiment was conducted in which gut microbiota of donor voles from the LG, MG, and HG (Experiment 2) was transplanted into individually housed voles as recipient voles (for more details, see below and Figure 4a). Specifically, 4‐month‐old male voles (*n* = 32) were selected and randomly assigned to four groups: control group (Con, *n* = 8), FMT group from the LG (T‐L, *n* = 8), FMT group from the MG (T‐M, *n* = 8), and FMT group from the HG (T‐H, *n* = 8). The recipient voles were raised in individual cages for 2 weeks of acclimation before the FMT experiment was conducted. After the FMT experiment, voles were sacrificed, and their feces, serum, tissues, and organs were collected immediately for further analysis.

*Experiment 4*: To test whether the effects of high‐density crowding stress on aging‐related processes could be alleviated, the effects of an anti‐inflammatory intervention were examined by administering butyric acid (for improving intestinal health through the anti‐inflammatory activity) and the effects of a crowding relief intervention, that is, shifting voles from the high‐density condition (six voles per cage) to low density condition (single vole per cage), on aging‐related processes (Figure 6a). 4‐month‐old male voles were selected and randomly assigned to six cages with six voles per cage, and they were raised for 8 weeks in the laboratory environment. By the end of the experiment, voles (from each cage) were randomly divided into three groups, namely the high‐density butyric acid feeding group (HB, *n* = 12), the high‐density relief group (HR, *n* = 8), and the high‐density continuation group (HC, *n* = 12). The butyrate solution was fed once a day (200 µL each time) for 4 successive weeks. The butyric acid was fed by gavage, and the dose level was 500 mg kg^−1^. After the experiment, voles were sacrificed, and their serum, tissues, and organs were collected immediately for further analysis.

### Sample Collection

Fecal pellet samples were collected on the day before the end of the experiment and were used for microbial analysis. Voles were placed in a sterile cage to defecate, and their fresh feces were collected and stored in a sterile centrifuge tube. At the end of the experiment, all experimental animals were sacrificed using CO_2_ between 9 and 11 AM. Before dissection, blood samples were obtained from voles through retro‐orbital bleeding, and the samples were then centrifuged at 4 °C (1500 rpm) for 30 min. Organs and tissues were immediately collected, rinsed with saline, and blot‐dried on a filter paper, and weighed (accurately to 0.001 g). All samples were stored at −80 °C. The serum CORT level, aging‐related marker concentrations, and oxidation levels of the organs were measured (see below).

### Fecal Microbiota Transplantation

Six to nine fresh feces pellets (about 200 mg) collected from donors (voles in LG, MG, and HG in Experiment 2,) were diluted in 2 mL of physiological saline and centrifuged (500 × *g* for 1 min) and the supernatant was used for FMT. Before FMT, 32 voles were treated with an antibiotic cocktail (containing 100 µg mL^−1^ neomycin, 50 µg mL^−1^ streptomycin, and 100 U/mL penicillin) for 7 days (100 µL per day) as described previously.^[^ ^32^ ^]^ After that, the FMT group was fed 100 µL of suspension, and the control group was fed 100 µL of normal saline. The solution was administered to voles by oral gavage once every 3 days for 4 weeks. During the 4 weeks, all recipient voles were fed with the same food as the control group.

### Metagenomic Sequencing and Preprocess

DNA in the feces sample was extracted using the Tiangen kit (#DP328, Tiangen Company, Beijing, China) according to the manufacturer's instructions. About 2 µg DNA per sample was prepared. Sequence libraries were generated using NEBNextR Ultra DNA library prep kit for Illumina (NEB, USA). The libraries were sequenced on the Illumina NovaSeq 6000 platform (insert size 350 bp, read length 150 bp). The low‐quality sequences were discarded, and the high‐quality sequences were assembled using SOAP denovo version 2.04 (http://soap.genomics.org.cn/soapdenovo.html).

### Measurement of Protein Expression by Western Blot

The brain (for detecting p‐p53 and p21), small intestine (for detecting claudin‐1), and liver (for detecting p‐p53, p21, p‐NF‐*κ*B p65, and COX‐2) (≈0.1 g) were homogenized in RIPA buffer and cleared by centrifugation, according to the standard techniques. Western blots of whole‐tissue lysates were probed with primary antibodies against p‐p53, p21, p‐NF‐*κ*B p65, COX‐2 (p‐p53: WL02504; p21: WL0362; claudin‐1: WL03073; p‐NF‐*κ*B p65: WL02169; COX‐2: WL01750; Wanleibio Co., Ltd.), and *β*‐actin (WL01372; Wanleibio Co., Ltd.). The secondary antibody was peroxidase‐conjugated goat anti‐rabbit IgG‐HRP (WLA023; Wanleibio Co., Ltd.). Protein markers (26616; Fermentas, Canada) were added on both sides of each gel to verify bands. The PVDF membranes were detected by enhanced chemoluminescence (WLA003; Wanleibio Co., Ltd.). Bands were analyzed using Gel‐Pro‐Analyzer Software, normalized to *β*‐actin, and expressed as relative units.

### Measurement of Relative Telomere Length by Real‐Time Quantitative PCR

The impacts of housing density were assayed on relative telomere length in the liver and brain. Tissue genomic DNA extraction kit (DP1902, BioTeke Corporation, China) was used to extract DNA from the brain and liver. AUV spectrophotometer NANO 2000 (Thermo, USA) was used to determine the concentration of DNA in each sample. RT‐qPCR analysis was carried out as follows: the DNA samples (1 µL) were used as a template for the subsequent PCR reaction using gene‐specific primers (Tel F: CGGTTTGTTTGGGTTTGGGTTTGGGTTTGGGTTTGGGTT; Tel R: GGCTTGCCTTACCCTTACCCTTACCCTTACCCTTACCCT; 36B4 F: TCTTCGACTAATCCCGCCAA; 36B4 R: CAAAGTAAACAGTAGCGGTTTTGC). The final reaction volume of 20 µL contained 10 µL of 2× Taq PCR MasterMix (PC1150, Solarbio), 1 µL of forward primer and reverse primer (final concentration 0.5 µL per primer), and 8 µL ddH2O. RT‐qPCR was performed using Exicycler 96 (BIONEER, Korea). After an initial polymerase activation step at 95 °C for 60 s, amplification was followed by 40 cycles (95 °C for 5 s, 55 °C for 30 s, and 72 °C for 30 s). The reaction was finished by the built‐in melting curve. All samples were quantified for relative quantity of gene expression by using 36B4 expression as an internal standard. The gene expression fold change was normalized to the control sample and processed by the 2^−ΔΔCT^ method using 36B4 as an internal control. Significant differences in expression of 36B4 between samples treated with exercise and detraining were not observed.

### Measurement of Serum Corticosterone, Tumor Necrosis Factor‐*α*, and Interleukin‐10 Content

The concentration of CORT in vole's serum was measured by a commercial kit (CEA540Ge 96T). The minimum detection concentration of this kit was 2.60 ng mL^−1^, the intra‐batch difference was less than 10%, and the inter‐batch difference was less than 12%. The content of TNF‐*α* and IL‐10 in voles’ serum was measured by mice commercial kit (EK282/3‐01, EK210/4‐04). The intra‐assay coefficients of variation were 5.1% (TNF‐*α*) and 3.7% (IL‐10), and the inter‐assay coefficients of variation were 6.6% (TNF‐*α*) and 4.1% (IL‐10).

### Measurement of Oxidative Damage and Antioxidant Levels

The level of 8‐OHdG and 4‐HNE, and activity of SOD and CAT in liver and brain tissues were detected to assess the oxidative damage and antioxidant levels in Brandt's voles. A commercial kit was used to measure 8‐OHdG to measure the level of DNA damage (CEA660Ge 96T). The intra batch difference was less than 10% and the inter batch difference was less than 12%. A commercial kit was used to measure 4‐HNE to measure the level of lipid damage (CSB‐E13412m, CUSABIO). Commercial kits were used to measure SOD to measure antioxidant levels (WLA110, wanleibio). Commercial kits were used to measure CAT to measure antioxidant levels (BC0205, Solarbio).

### The Y‐Maze Test

The Y‐maze apparatus comprised a white plastic maze with three arms (40 cm in length, 30 cm in height, and 8 cm in width) that intersect at 120°. The back wall of each arm was marked with different colored shapes. The voles began the test after a 12‐h fasting period. First, only two arms were opened and placed food in one arm, called the “food” arm. Voles were placed at one end of the “beginning” arm for 5 min to adapt and learn the location of the food and association with a colored shape marker. Voles were then removed from the Y‐maze for 1 h. Next, in the formal test, the food was removed and the third arm (“novel” arm) was opened, but did not change the markings on the walls. Thus, this method determined whether the animal could find the original location of the food by using only the markings on the wall of the enclosure. Animals were allowed to move freely for 5 min, and the number of times the vole visited the “food” arm and the “novel” arm and the cumulative time spent in the “food” arm and the “novel” arm were calculated.

### Statistical Analysis

The Kaplan–Meier method in the “survfit” function of the “survival” package of R software was used to fit the survival curve. The “survdiff” function was used to test the difference in the overall survival probability among the density treatment groups. Multiple comparisons were performed to evaluate the difference in between‐group survival probability by using the “pairwise_survdiff” function in the “survminer” package. Given the independence of individuals within the same cages, a linear mixed model was used to determine the differences in levels of CORT, oxidative damage, and protein expression levels and relative telomere length in brain and liver tissues between different treatments in Experiment 2. Rearing cages were treated as a random factor. All data analyses were conducted in R (version 4.1.1) with the “lme4” package. Multiple comparisons between treatment groups were performed using the “emmeans” package and the “emmeans” function. One‐way analysis of variance was used to determine whether FMT affected the above‐mentioned physiological aging indicators in Experiment 3. Pairwise multiple comparisons were performed to evaluate the between‐group difference by using Tukey's test. The model residuals were examined to determine whether they were normally distributed. If not, the data were statistically analyzed by the Kruskal–Wallis test. The Student's *t* test was used to determine whether the two interventions affected stress and physiological aging‐related indicators in Experiment 4. The false discovery rate approach was used to adjust the *p*‐value in Experiment 4. Permutational multivariate analysis of variance of gut microbial composition based on the Bray–Curtis index was performed using the “adonis” function in the “Vegan” package. Correlation between microbial species relative abundance dissimilarity matrix and physiological parameter distance matrix was determined by the Mantel test. The functions of genes of gut microbiota were profiled on the basis of the KEGG and CAZy databases by using DIAMOND software.

## Conflict of Interest

The authors declare no conflict of interest.

## Author Contributions

X.M.X. and G.L.L. contributed equally to this work. Z.B.Z. and X.M.X. designed the study; X.M.X. worked on the methodology; X.M.X., D.Z., and H.Z. performed investigation; X.M.X. and G.L.L. worked on visualization; Z.B.Z., G.L.L., and G.‐H.L. supervised this work; X.M.X., G.L.L., and Z.B.Z. wrote the original draft; X.M.X., G.L.L., and Z.B.Z. reviewed and edited the writing.

## Supporting information

Supporting Information

Supporting Information

## Data Availability

The data that support the findings of this study are available from the corresponding author upon reasonable request.
