# Supplementary material for: Gut Microbiota is Associated with Aging‐Related Processes of a Small Mammal Species under High‐Density Crowding Stress
Source: Adv Sci (Weinh). 2023 Mar 25;10(14):2205346. doi: 10.1002/advs.202205346 (PMC10190659; doi:10.1002/advs.202205346)
Supplement: Supplementary file 1 — Supporting Information [file ADVS-10-2205346-s002.pdf]

Supporting Information

Gut microbiota is associated with aging-related processes of a small mammal species under high-density crowding stress

Xiaoming Xu[1, 2, 3], *Guoliang Li*[1, 2], *Da Zhang*[1, 2, 3], *Hanyi Zhu*[1, 2, 3], *Guanghui-Liu* [4, 5, 6] ， *Zhibin Zhang*[1, 2] *

1 State Key Laboratory of Integrated Management of Pest Insects and Rodents, Institute of Zoology, Chinese Academy of Sciences, Beijing 100101, China.

2 CAS Center for Excellence in Biotic Interactions, University of Chinese Academy of Sciences, Beijing 100049, China.

3 University of Chinese Academy of Sciences, Beijing 100049, China.

4 Institute for Stem cell and Regeneration, CAS, Beijing 100049, China.

5 State Key Laboratory of Membrane Biology, Institute of Zoology, Chinese Academy of Sciences, Beijing 100101, China.

6 Beijing Institute for Stem Cell and Regenerative Medicine, Beijing 100101, China.

* To whom correspondence is addressed: Email: zhangzb@ioz.ac.cn

This file includes:

Supplementary Text
Figs. S1 to S11
Tables S1 to S6
Measurement of resting metabolic rate (RMR)
Measurement of CD38, occludin and sirt1 protein expression by western blot
Measurement of NAD^+ and protein carbonyl (PC)
Measurement of Short-chain fatty acids (SCFAs)

Supplementary Text

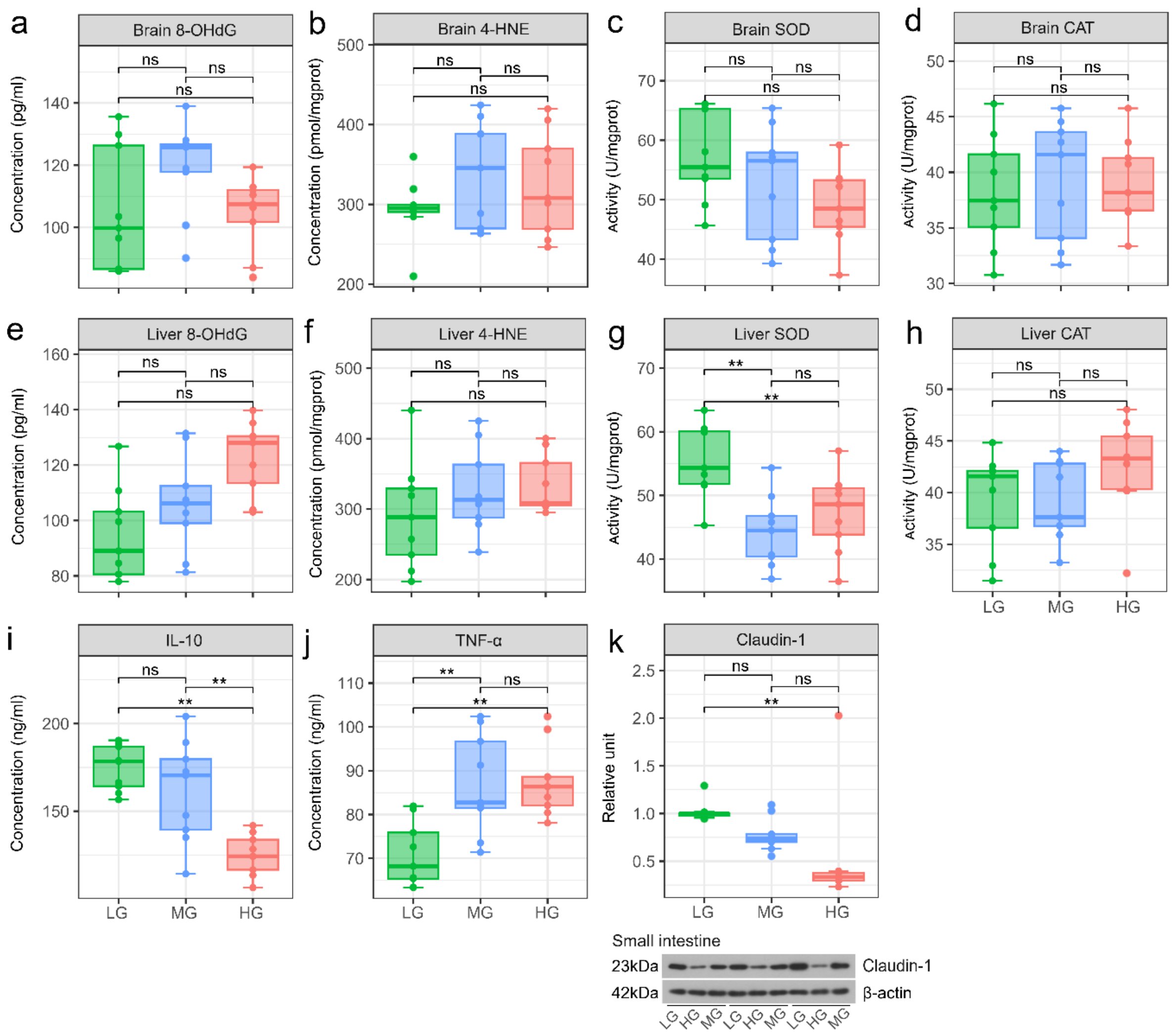

Figure S1. Effects of housing densities on related oxidative stress, inflammation and claudin-1. a-d) 8-OHdG and 4-HNE levels, and SOD and CAT activity levels in brain. e-h) 8-OHdG and 4-HNE levels, and SOD and CAT activity levels in liver. i) and j) Serum TNF-α and IL-10 levels. k) Claudin-1 expression in small intestine. For box plot, centerline: median; box limits: upper and lower quartiles; whiskers: two lines that go from the minimum to the lower quartile and then from the upper quartile to the maximum. Linear mixed model was used to determine the differences between groups, with groups as fixed factors and different cages as random factors. The "emmeans" function were used to perform multiple comparisons test. * $p < 0.05$, ** $p < 0.01$, ns, not statistically significant. LG, low-density group, $n = 9$; MG, medium-density group, $n = 9$; HG, high-density group, $n = 9$.

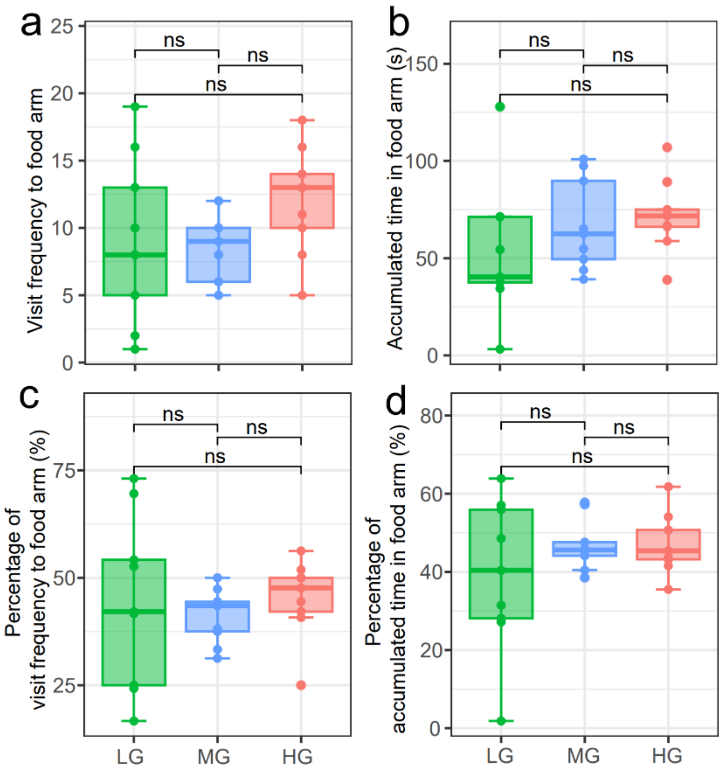

Figure S2. Effects of housing densities on cognitive function. a) Visit frequency to food arm. b) Accumulated time in food arm. c) Percentage of visit frequency to food arm. Visit frequency to food arm / (visit frequency to food arm + visit frequency to novel arm) × 100%. d) Percentage of accumulated time in food arm. Accumulated time in food arm / (accumulated time in food arm + accumulated time in novel arm) × 100%. For box plot, centerline: median; box limits: upper and lower quartiles; whiskers: two lines that go from the minimum to the lower quartile and then from the upper quartile to the maximum. One-way analysis of variance was used to determine the differences between groups, Tukey test was used for post hoc. * $p < 0.05$, ** $p < 0.01$, ns, not statistically significant. LG, low-density group, $n = 9$; MG, medium-density group, $n = 9$; HG, high-density group, $n = 9$.

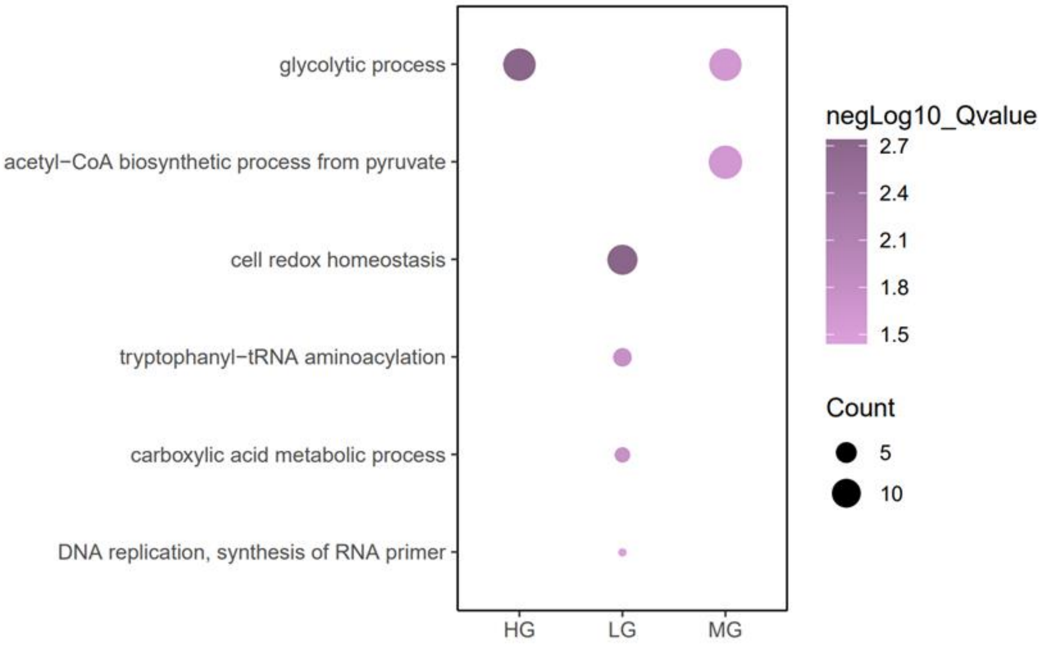

Figure S3. Differences in the biological processes in the GO enrichment analysis between the LG, MG, and HG. The differential KO pathway (from Table S2) was transformed into GO enrichment through the DAVID website. LG, low-density group, n = 9; MG, medium-density group, n = 10; HG, high-density group, n = 11.

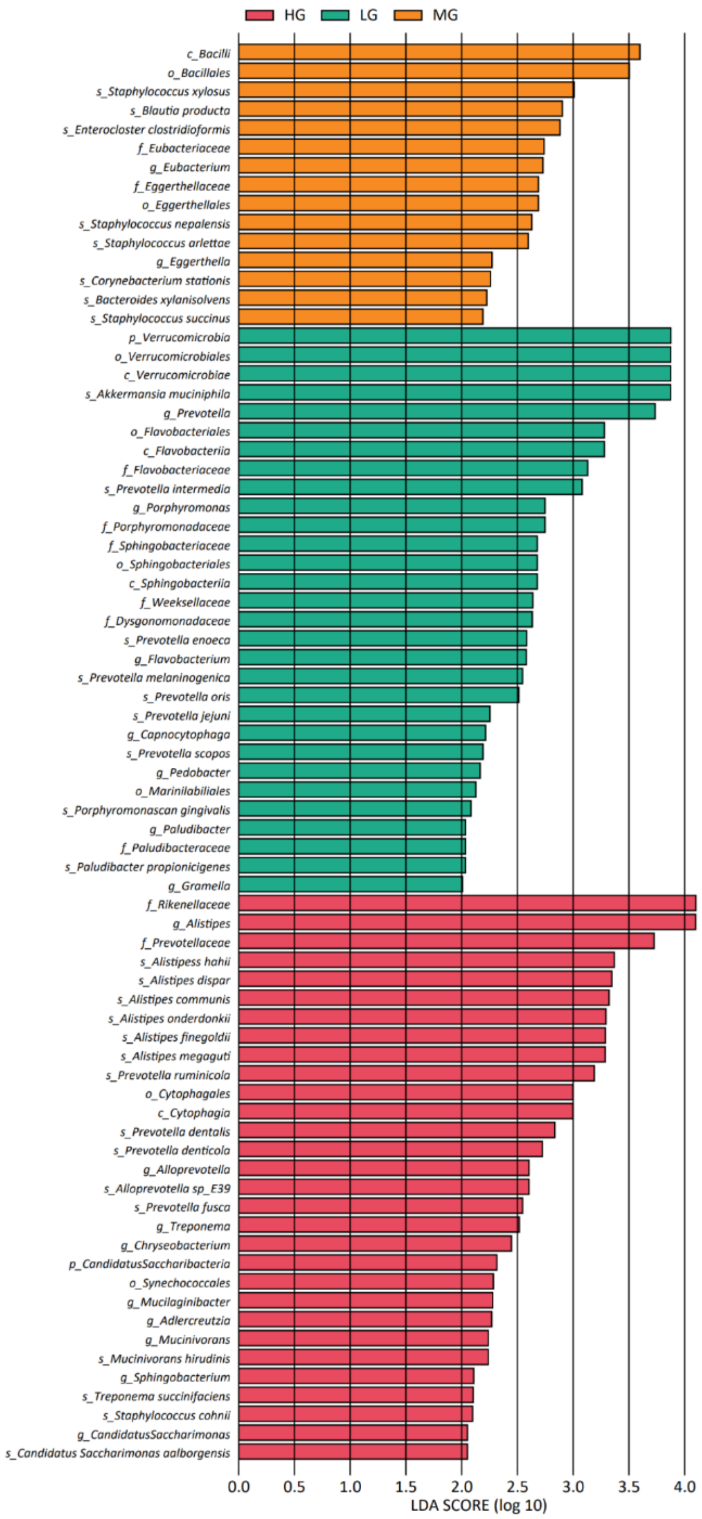

Figure S4. Differences in bacterial abundance between the LG, MG, and HG. The figure shows the distribution of linear discriminant analysis (LDA) values of different taxon. The color represents the corresponding group, and the length of the histogram represents the contribution of different genera (LDA score > 2). LG, low-density group, n = 9; MG, medium-density group, n = 9; HG, high-density group, n = 9.

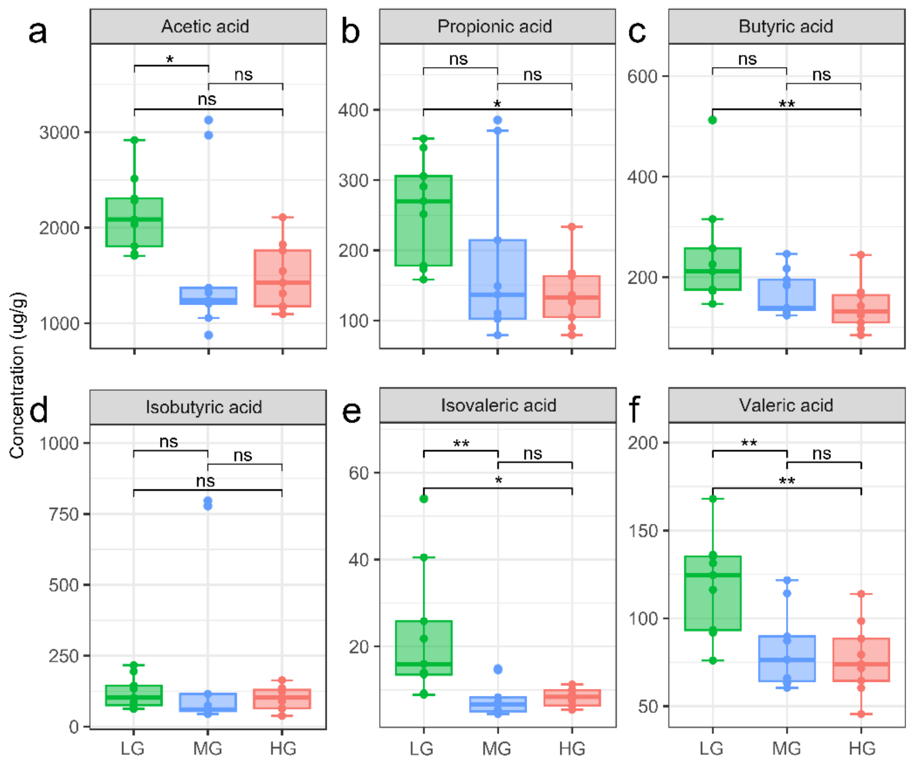

Figure S5. Effects of housing densities on the concentration of short chain fatty acids in feces. a) Acetic acid. b) Propionic acid. c) Butyric acid. d) Isobutyric acid. e) Isovaleric acid. f) Valeric acid. For box plot, centerline: median; box limits: upper and lower quartiles; whiskers: two lines that go from the minimum to the lower quartile and then from the upper quartile to the maximum. Kruskal-Wallis statistic was used to determine the differences between groups, Dunn's test was used for post hoc. * $p < 0.05$, ** $p < 0.01$, ns, not statistically significant. LG, low-density group, $n = 9$; MG, medium-density group, $n = 9$; HG, high-density group, $n = 9$.

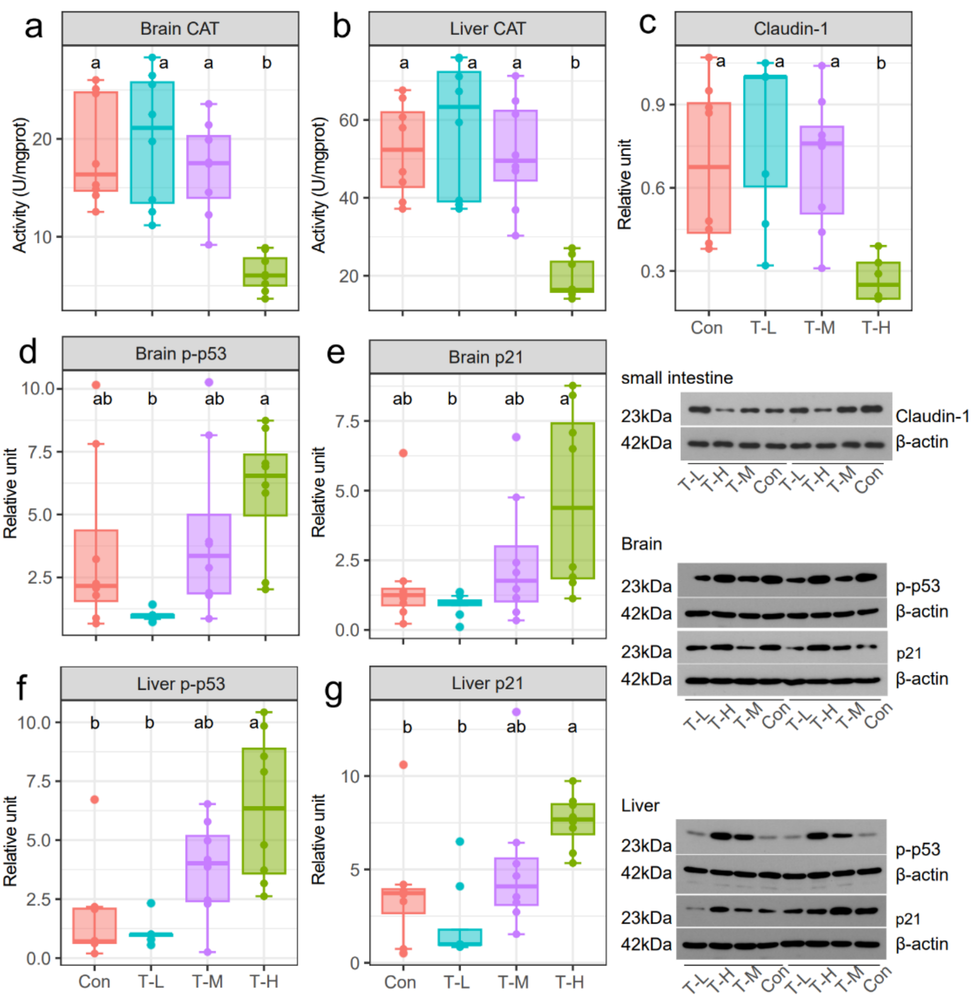

Figure S6. Effects of FMT of donor voles in the three density groups on CAT, claudin-1, p-p53, and p21 expression levels in recipient voles. a) and b) CAT activity level in brain and liver. c) Claudin-1 expression level in small intestine. d) and e) p-p53 and p21 expression levels in brain. f) and g) p-p53 and p21 expression levels in in liver. For box plot, centerline: median; box limits: upper and lower quartiles; whiskers: two lines that go from the minimum to the lower quartile and then from the upper quartile to the maximum. T-L, Recipient voles with FMT from low-density group (LG), *n* = 8; T-M, Recipient voles with FMT from medium-density group (MG), *n* = 8; T-H, Recipient voles with FMT from high-density group (HG), *n* = 8; Con, Voles with saline, *n* = 8. One-way analysis of variance was used to determine the difference between groups, and Tukey test was used for post hoc. Bars that do not share the same letter are significantly different from one another.

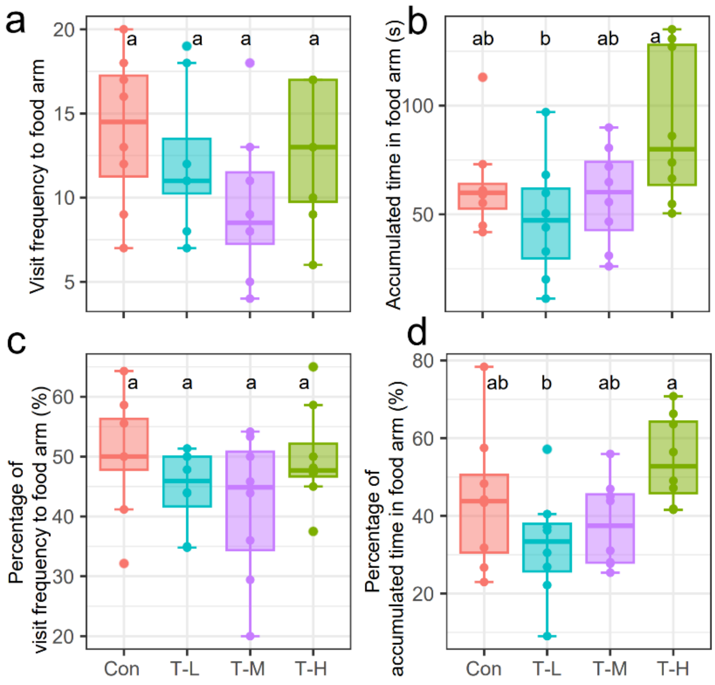

Figure S7. Effects of FMT of donor voles in the three density groups on the cognitive function of recipient voles. a) Visit frequency to food arm. b) Accumulated time in food arm. c) Percentage of visit frequency to food arm. Visit frequency to food arm / (visit frequency to food arm + visit frequency to novel arm) × 100%. d) Percentage of accumulated time in food arm. Accumulated time in food arm / (accumulated time in food arm + accumulated time in novel arm) × 100%. For box plot, centerline: median; box limits: upper and lower quartiles; whiskers: two lines that go from the minimum to the lower quartile and then from the upper quartile to the maximum. One-way analysis of variance was used to determine the differences between groups, Tukey test was used for post hoc. * $p < 0.05$, ** $p < 0.01$, ns, not statistically significant. Bars that do not share the same letter are significantly different from one another. T-L, Recipient voles with FMT from low-density group (LG), $n = 8$; T-M, Recipient voles with FMT from medium-density group (MG), $n = 8$; T-H, Recipient voles with FMT from high-density group (HG), $n = 8$; Con, Voles with saline, $n = 8$.

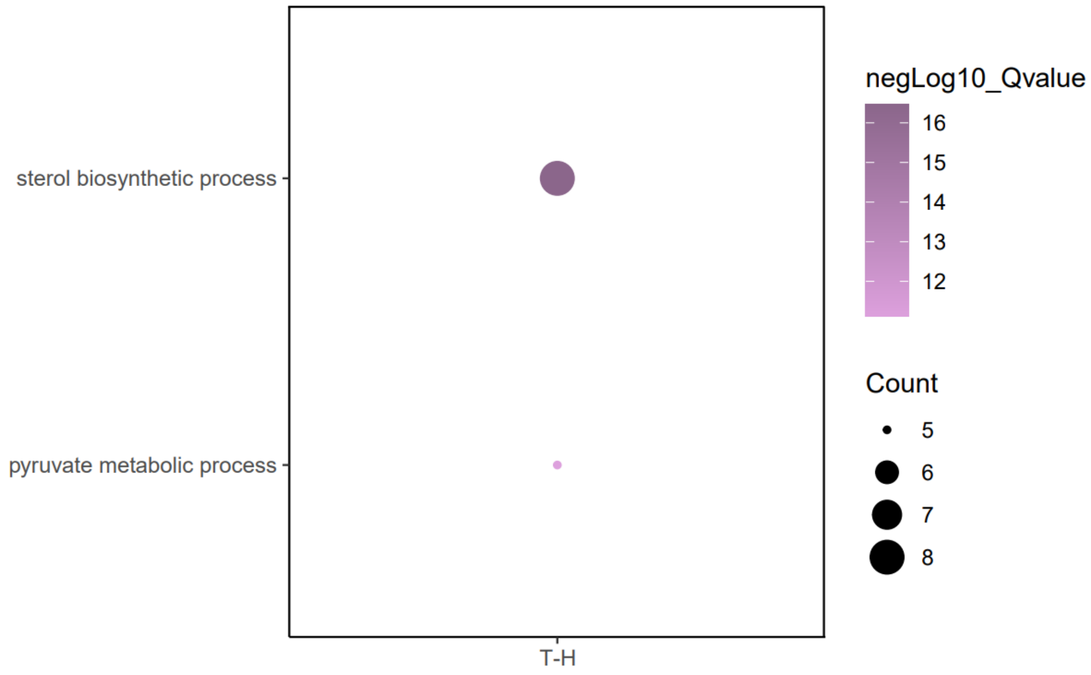

Figure S8. Differences in the biological processes in the GO enrichment analysis between the T-L, T-M, and T-H.

The differential KO pathway (from Table S4) was transformed into GO enrichment through the DAVID website. T-L, Recipient voles with FMT from low-density group (LG), *n* = 8; T-M, Recipient voles with FMT from medium-density group (MG), *n* = 8; T-H, Recipient voles with FMT from high-density group (HG), *n* = 8; Con, Voles with saline group, *n* = 8.

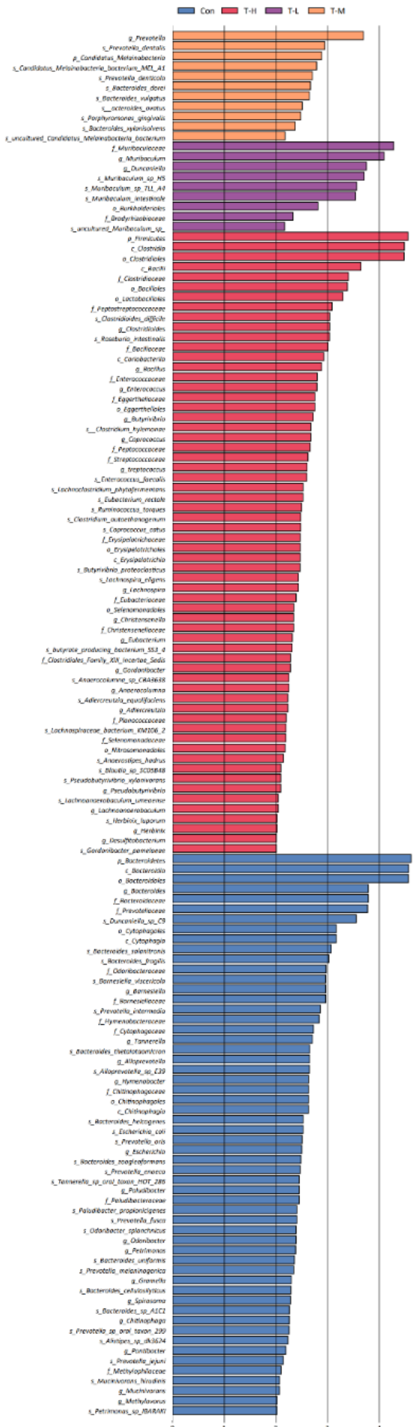

Figure S9. Differences in bacterial abundance between the Con, T-L, T-M and T-H. The figure shows the distribution of linear discriminant analysis (LDA) values of different taxon. The color represents the corresponding group, and the length of the histogram represents the contribution of different genera (LDA score > 2). T-L, Recipient voles with FMT from low-density group (LG), *n* = 8; T-M, Recipient voles with FMT from medium-density group (MG), *n* = 8; T-H, Recipient voles with FMT from high-density group (HG), *n* = 8; Con, Voles with saline group, *n* = 8.

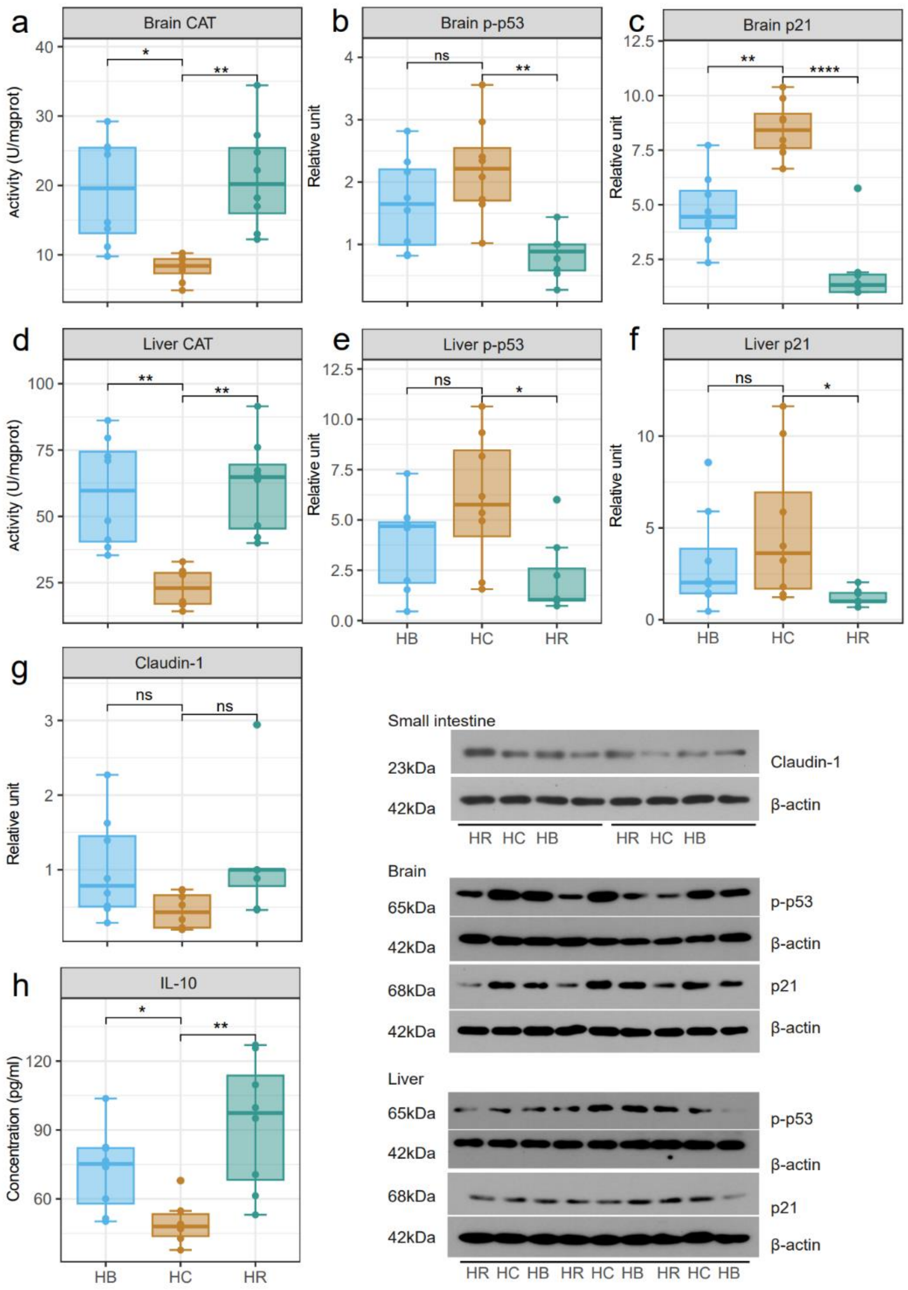

Figure S10. Effects of different intervention methods on CAT, claudin-1, p-p53, p21 and IL-10 of HG voles. a-c) CAT activity level and p-p53 and p21 expression levels in brain. d-f) CAT activity level and p-p53 and p21 expression levels in liver. g) Claudin-1 expression level in small intestine. h) Serum IL-10 level. For box plot, centerline: median; box limits: upper and lower quartiles; whiskers: two lines that go from the minimum to the lower quartile and then from the upper quartile to the maximum. HB, High-density butyric acid feeding group, $n = 8$; HR, High-density relief group, $n = 8$; and HC, High-density continuation group (i.e., no high-density relief), $n = 8$. The statistical analysis in a-h) was performed using two-tailed unpaired *t*-test. HC was included as control. * $p < 0.05$, ** $p < 0.01$, ns: not statistically significant.

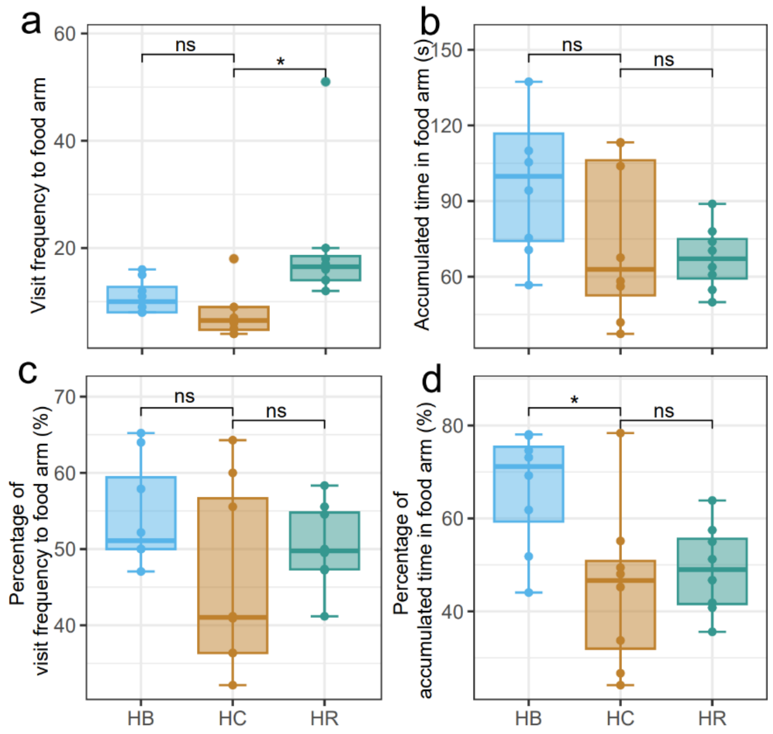

Figure S11. Effects of different intervention methods on cognitive function of HG voles. a) Visit frequency to food arm. b) Accumulated time in food arm. c) Percentage of visit frequency to food arm. Visit frequency to food arm / (visit frequency to food arm + visit frequency to novel arm) × 100%. d) Percentage of accumulated time in food arm. Accumulated time in food arm / (accumulated time in food arm + accumulated time in novel arm) × 100%. For box plot, centerline: median; box limits: upper and lower quartiles; whiskers: two lines that go from the minimum to the lower quartile and then from the upper quartile to the maximum. The statistical analysis in a) was performed using Mann-Whitney U test. The statistical analysis in b-d) was performed using two-tailed unpaired *t*-test. HC was included as control. $*p < 0.05$, $**p < 0.01$, ns: not statistically significant. HB, High-density butyric acid feeding group, $n = 8$; HR, High-density relief group, $n = 8$; and HC, High-density continuation group (i.e., no high-density relief), $n = 8$.

Table S1.

The CAZy pathway analysis with significant differences among the three groups in Experiment 2

CAZy	HG	LG	MG	Enrichment
GH36	0.049	0.000	0.006	HG
GH73	11.249	8.400	9.082	HG
GH33	127.470	108.350	134.330	LG
GT21	3.145	1.600	3.241	LG
CBM20	29.810	25.261	15.446	MG
GH16	29.822	31.429	43.470	MG
GH59	1.398	1.161	2.768	MG
GH65	257.308	262.179	204.091	MG
GT36	126.547	122.039	154.075	MG
GT51	665.297	653.769	581.202	MG

Numbers in the table represent the relative abundance of each sample. The differences between the functional genomes shown in the table are significant ($p < 0.05$).

Table S2.

The KO pathway analysis with significant differences among the three groups in Experiment 2

KO	HG	LG	MG	Enrichment
K00016	69.711	47.739	70.185	LG
K00046	0.040	0.183	0.032	LG
K00057	211.166	192.918	192.115	HG
K00058	512.641	442.916	542.008	LG
K00097	135.710	115.583	115.854	HG
K00162	12.118	10.237	18.416	MG
K00183	2.498	1.083	2.145	LG
K00244	318.203	256.325	259.215	HG
K00588	165.777	147.865	145.846	HG
K00674	21.275	11.763	19.952	LG
K00759	301.137	271.801	274.904	HG
K00919	181.957	162.980	182.876	LG
K00969	642.623	576.078	636.017	LG
K00996	61.727	62.854	48.785	MG
K01151	176.424	137.563	161.174	LG
K01216	33.694	35.128	19.146	MG
K01405	0.648	0.099	0.132	HG
K01420	67.963	71.798	52.083	MG
K01465	414.741	386.196	386.784	HG
K01623	6.367	2.791	3.159	HG
K01736	206.745	172.359	200.074	LG
K01867	322.458	280.947	312.698	LG
K01925	265.391	235.271	260.804	LG
K02043	0.285	0.044	0.020	HG

The KO pathway analysis with significant differences among the three groups in Experiment 2 (continued)

KO	HG	LG	MG	Enrichment
K09761	180.118	165.092	181.265	LG
K10914	98.241	99.226	75.516	MG
K10966	5.918	3.467	3.272	HG
K11063	0.423	0.446	0.944	MG
K11261	7.458	3.763	7.591	LG
K11320	0.016	0.205	0.000	LG
K12060	0.000	0.006	0.000	LG
K12146	10.715	21.756	10.912	LG
K12266	11.739	22.256	11.109	LG
K12339	8.219	3.577	3.873	HG
K12688	2.015	2.825	1.851	LG
K13310	109.620	114.916	85.911	MG
K13726	0.648	0.099	0.132	HG
K13810	1.994	1.067	2.159	LG
K13811	3.008	2.064	5.375	MG
K13829	472.355	412.060	460.706	LG
K14550	0.012	0.143	0.031	LG
K15173	0.000	0.159	0.011	LG
K15460	179.859	161.052	180.458	LG
K15525	0.000	0.000	0.121	MG
K15527	8.213	3.185	8.380	LG
K16079	11.756	6.477	6.731	HG
K16193	462.962	420.539	474.513	LG
K16743	0.002	0.000	0.114	MG
K16751	0.000	0.135	0.000	LG
K16905	7.275	15.180	14.643	HG
K17630	0.016	0.130	0.020	LG
K17758	170.029	150.163	173.601	LG
K17759	163.822	142.283	162.469	LG
K17900	0.009	0.145	0.028	LG
K18299	0.814	1.734	0.830	LG
K18330	46.019	45.208	60.754	MG
K18354	5.316	2.286	2.544	HG
K18702	4.529	2.333	2.681	HG
K18914	0.215	0.049	0.035	HG
K18930	81.617	55.185	77.084	LG
K19167	21.956	13.131	21.260	LG
K19302	278.333	278.421	229.617	MG
K19340	0.006	0.008	0.197	MG
K19516	0.071	0.321	0.046	LG
K19551	5.149	5.358	2.109	MG
K19953	0.000	0.055	0.000	LG
K20236	0.016	0.130	0.004	LG
K20306	44.821	52.770	28.634	MG
K20338	16.735	27.608	15.069	LG
K20344	233.921	341.471	205.876	LG
K21148	7.706	3.408	3.594	HG
K21268	0.019	0.001	0.000	HG
K22340	43.867	43.640	58.068	MG
K22476	3.285	2.821	7.374	MG
K19551	5.149	5.358	2.109	MG

Numbers in the table represent the relative abundance of each sample. The differences between the functional genomes shown in the table are significant ($p < 0.05$).

Table S3.

The indicators and their statistical values in Experiment 3

Index	*F* value	value					
		T-L vs. T-M	T-L vs. T-H	T-L vs. Con	T-M vs. T-H	T-M vs. Con	T-H vs. Con
Brain SOD	*F* (3, 28) = 21.26	0.724	<0.0001	0.0108	<0.0001	0.1136	0.0024
Brain CAT	*F* (3, 28) = 14.91	0.7284	<0.0001	0.8129	0.0002	0.9987	0.0001
Brain relative telomere length	*F* (3, 28) = 13.57	0.7455	<0.0001	0.7091	0.0003	>0.9999	0.0003
Brain 8-OHdG	*F* (3, 28) = 27.01	0.7198	<0.0001	0.3796	<0.0001	0.9388	<0.0001
Brain 4-HNE	*F* (3, 28) = 25.30	0.8729	<0.0001	0.329	<0.0001	0.7671	<0.0001
Liver COX-2	*F* (3, 28) = 6.579	0.9631	0.0041	>0.9999	0.0136	0.9683	0.0044
Liver 8-OHdG	*F* (3, 28) = 23.61	0.7919	<0.0001	0.5002	<0.0001	0.9604	<0.0001
Liver 4-HNE	*F* (3, 28) = 28.62	0.9	<0.0001	0.4759	<0.0001	0.8686	<0.0001
Liver relative telomere length	*F* (3, 28) = 10.78	0.5291	<0.0001	0.6159	0.0024	0.999	0.0017
Liver p-NF-κB p65	*F* (3, 28) = 15.50	0.6042	<0.0001	0.7849	0.0001	0.7849	<0.0001
Small intestine claudin-1	*F* (3, 28) = 7.845	0.7565	0.0006	0.7268	0.0075	>0.9999	0.0085
Serum Cort	*F* (3, 28) = 53.07	0.3933	<0.0001	0.5876	<0.0001	0.9873	<0.0001
Serum TNF-α	*F* (3, 28) = 41.28	0.6814	<0.0001	0.0561	<0.0001	0.4173	<0.0001
Serum IL-10	*F* (3, 24) = 6.572	0.4045	0.0016	0.0453	0.0345	0.622	0.2404
Liver SOD	*F* (3, 28) = 21.26	0.7240	<0.0001	0.0108	<0.0001	0.1136	0.0024
Liver CAT	*F* (3, 28) = 14.91	0.7284	<0.0001	0.8129	0.0002	0.9987	0.0001
Brain p-p53		0.0580	0.0021	0.3090	>0.9999	>0.9999	0.6222
Brain p21		0.3941	0.0056	>0.9999	0.8541	>0.9999	0.1056
Liver p21		0.5572	0.0021	>0.9999	0.3494	>0.9999	0.0496
Liver p-p53	*F* (3, 28) = 9.588	0.0748	0.0002	0.5584	0.0748	0.1332	0.0010
Visit frequency to food arm	*F* (3, 28) = 1.502	0.6670	0.8172	0.7827	0.5529	0.2611	0.8172
Accumulated time in food arm	*F* (3, 28) = 3.496	0.7042	0.0260	0.6022	0.1250	0.7046	0.2185
Percentage of visit frequency to food arm	*F* (3, 28) = 1.549	0.7796	0.6814	0.6814	0.3958	0.3958	0.9364
Percentage of accumulated time in food arm	*F* (3, 28) = 3.689	0.6321	0.0219	0.3586	0.1172	0.6231	0.3776

Note: $p < 0.05$ indicates a significant difference. Kruskal-Wallis statistic was used to determine the differences of brain p-p53, brain p21, and liver p21 between groups, Dunn's test was used for post hoc.

Table S4.

The CAZy pathway analysis with significant differences among the three groups in Experiment 3

CAZy	T-H	T-L	T-M	Enrichment
GH115	8603.393	11496.871	11330.394	HG
GT3	3328.567	4477.854	4471.956	HG
GT30	1837.082	2548.855	2688.706	HG
GH105	7477.211	10426.149	11092.340	HG
CE11	2482.668	3302.453	3405.284	HG
GT9	2095.504	2870.693	2945.096	HG
CE8	10594.309	13960.586	14659.682	HG
GH97	9560.775	13109.982	13242.442	HG
GH133	4565.776	5927.862	5883.801	HG
GH57	3573.552	4473.488	4601.568	HG
GH28	9581.198	13866.281	14697.150	HG
GH20	6548.434	9750.037	9981.903	HG
GT19	2820.617	3864.405	3772.952	HG
GH25	10352.193	8394.724	10210.330	LG
GH55	280.000	404.091	317.387	LG
GH78	7841.181	8896.915	11493.578	MG
GT23	153.625	124.000	354.000	MG
GT51	10458.808	10352.350	11548.058	MG
CBM67	5104.283	5708.427	7248.726	MG

Table S5.

The KO pathway analysis with significant differences among the three groups in Experiment 3

KO	T-H	T-L	T-M	Enrichment
k04722	87.250	221.375	203.250	HG
k00600	44161.024	51926.135	56126.368	HG
k00565	1485.235	2149.684	2113.369	HG
k00531	12982.586	17161.527	17495.361	HG
k00311	1655.373	2130.090	2288.011	HG
k00280	17874.789	20867.295	21805.320	HG
k00590	2413.127	2902.136	2847.800	HG
k00908	5818.921	6566.561	6992.444	HG
k00430	12826.209	15230.026	15176.790	HG
k00790	24643.102	28279.704	30260.890	HG
k04022	24643.102	28279.704	30260.890	HG
k00020	72108.095	80791.878	84371.449	HG
k04974	5109.246	6647.108	7038.018	HG
k00460	36813.439	41536.342	42298.740	HG
k00940	25263.723	28994.873	29146.793	HG
k00604	5531.059	8374.412	8532.143	HG
k04142	14567.298	20034.018	20458.533	HG
k00471	16722.536	18767.252	19342.751	HG
k00540	27812.789	36744.492	37631.710	HG
k00591	1513.610	2149.059	2118.994	HG
k04614	2766.602	3932.629	3984.118	HG
k00510	2306.901	2823.614	2736.344	HG
k01053	1599.799	2120.647	2048.334	HG
k04210	2412.712	3081.447	3148.915	HG
k00627	3739.254	4788.050	4949.156	HG
k04620	3739.254	4788.050	4949.156	HG
k00511	60962.106	76277.480	81156.575	HG
k00785	2499.078	3243.814	3233.556	HG
k05133	5638.408	7406.496	7304.381	HG
k00603	13814.778	16360.369	17181.882	HG
k00592	1458.860	2119.434	2084.869	HG
k00562	9483.023	11091.700	11164.662	HG
k00950	6642.915	7983.095	8203.327	HG
k00534	6642.915	7983.095	8203.327	HG
k00130	12599.757	17020.355	16150.112	HG
k00362	5949.037	4921.627	5991.580	LG
k00622	2356.526	1508.873	2206.265	LG
k00621	2341.901	1501.123	2200.140	LG
k04141	10331.261	10750.202	12054.196	MG
k00533	1.750	1.250	8.375	MG
k05146	5270.037	5201.711	6520.520	MG
k05143	53.250	65.500	107.000	MG

Numbers in the table represent the relative abundance of each sample. The differences between the functional genomes shown in the table are significant ($p < 0.05$).

Table S6.

The indicators and their statistical values in Experiment 4

Index	Adjusted *P*-value HR vs. HC	Significance	Adjusted *P*-value HR vs. HB	Significance
Brain SOD	0.0030	**	0.0135	*
Brain CAT	0.0036	**	0.0135	*
Brain relative telomere length	0.0739	ns	0.0849	ns
Brain 8-OHdG	0.0022	**	0.0078	**
Brain 4-HNE	0.0022	**	0.0078	**
Liver COX-2	0.0057	**	0.0530	ns
Liver 8-OHdG	0.0022	**	0.0078	**
Liver 4-HNE	0.0022	**	0.0089	**
Liver relative telomere length	0.0648	ns	0.1750	ns
Liver p-NF-κB p65	0.0317	*	0.1138	ns
Small intestine claudin-1	0.0648	ns	0.0849	ns
Serum CORT	0.0014	**	0.0089	**
Serum TNF-α	0.0022	**	0.0135	*
Serum IL-10	0.0060	**	0.0296	*
Liver SOD	0.0030	**	0.0297	*
Liver CAT	0.0022	**	0.0078	**
Brain p-p53	0.0031	**	0.1904	ns
Liver p-p53	0.0221	*	0.1750	ns
Brain p21	0.0000	****	0.0069	**
Liver p21	0.0473	*	0.3190	ns

Note: $p < 0.05$ indicates a significant difference.

Measurement of Resting Metabolic Rate (RMR).

At a temperature of 30 °C ± 0.5, the oxygen consumption of voles was measured using the open-circuit respirometry system (TSE, German). The air flow rate is 0.8 L/min, and the data collection interval was 1 minutes. Oxygen consumption was measured for three hours. The average of two lowest consecutive readings was taken as the RMR.

Measurement of CD38, Occludin and Sirt1 Protein Expression by Western Blot

The brain (for detecting CD38 and occludin) and liver (for detecting CD38 and sirt1) (~0.1g) were homogenized in RIPA buffer and cleared by centrifugation, according to the standard techniques. Western blots of whole-tissue lysates were probed with primary antibodies against, CD38, occludin and sirt1 (CD38: WL02870; occludin: WL01996; sirt1: WL00599; Wanleibio Co., Ltd.) and β-actin (WL01372; Wanleibio Co., Ltd.). The secondary antibody was peroxidase-conjugated goat anti-rabbit IgG-HRP (WLA023; Wanleibio Co., Ltd.). Protein markers (26616; Fermentas, Canada) were added on both sides of each gel to verify bands. The PVDF membranes were detected by enhanced che-moluminescence (IPVH00010, Millipore). Bands were analyzed using Gel-Pro-Analyzer Software, normalized to β-actin, and expressed as relative units (RU).

Measurement of NAD^+ and Protein Carbonyl (PC)

The content of NAD^+ in the brain and liver is measured by a commercial kit (BC0315, Solabio). The levels of protein carbonyl were measured by a commercial kit (BC1275, Solabio).

Measurement of Short-Chain Fatty Acids (SCFAs)

We measured six SCFAs: acetic acid, propionic acid, butyric acid, isobutyric acid, valeric acid, and isovaleric acid from colonic feces. SCFAs were measured via high performance gas chromatography (GC) (Agilent 7890A; Agilent Technologies, Germany) with a GC autosampler and an FID system. Separations were performed in a 30 m × 0.25 mm × 0.25μm DB-WAX column (Agilent Technologies). In total, 99.998% hydrogen was used as carrier gas at a flow-rate of 1.0 mL/min. The system was operated at 250 °C. Injections were performed in the split less mode at 230 °C, and 0.5 μL for each injection. The oven temperature was programmed from 60 °C (1 min) at 5 °C/min to 200 °C and then from 200 °C at 10 °C/min to 230 °C. The total running time of each sample was 32 min.
